# In Situ Surface Fluorination of TiO_2_ Nanocrystals Reinforces Interface Binding of Perovskite Layer for Highly Efficient Solar Cells with Dramatically Enhanced Ultraviolet‐Light Stability

**DOI:** 10.1002/advs.202004662

**Published:** 2021-03-13

**Authors:** Wanpei Hu, Zhiling Wen, Xin Yu, Peisen Qian, Weitao Lian, Xingcheng Li, Yanbo Shang, Xiaojun Wu, Tao Chen, Yalin Lu, Mingtai Wang, Shangfeng Yang

**Affiliations:** ^1^ Hefei National Laboratory for Physical Sciences at Microscale CAS Key Laboratory of Materials for Energy Conversion Anhui Laboratory of Advanced Photon Science and Technology Department of Materials Science and Engineering University of Science and Technology of China Hefei 230026 China; ^2^ Institute of Solid State Physics Hefei Institutes of Physical Science Chinese Academy of Sciences Hefei 230031 China

**Keywords:** electron transport layer, fluorination, interface binding, perovskite solar cells, titanium oxide

## Abstract

Low‐temperature solution‐processed TiO_2_ nanocrystals (LT‐TiO_2_) have been extensively applied as electron transport layer (ETL) of perovskite solar cells (PSCs). However, the low electron mobility, high density of electronic trap states, and considerable photocatalytic activity of TiO_2_ result in undesirable charge recombination at the ETL/perovskite interface and notorious instability of PSCs under ultraviolet (UV) light. Herein, LT‐TiO_2_ nanocrystals are in situ fluorinated via a simple nonhydrolytic method, affording formation of Ti─F bonds, and consequently increase electron mobility, decrease density of electronic trap states, and inhibit photocatalytic activity. Upon applying fluorinated TiO_2_ nanocrystals (F‐TiO_2_) as ETL, regular‐structure planar heterojunction PSC (PHJ‐PSC) achieves a champion power conversion efficiency (PCE) of 22.68%, which is among the highest PCEs for PHJ‐PSCs based on LT‐TiO_2_ ETLs. Flexible PHJ‐PSC devices based on F‐TiO_2_ ETL exhibit the best PCE of 18.26%, which is the highest value for TiO_2_‐based flexible devices. The bonded F atoms on the surface of TiO_2_ promote the formation of Pb─F bonds and hydrogen bonds between F^−^ and FA/MA organic cations, reinforcing interface binding of perovskite layer with TiO_2_ ETL. This contributes to effective passivation of the surface trap states of perovskite film, resulting in enhancements of device efficiency and stability especially under UV light.

## Graphical abstract

In situ surface fluorination of TiO_2_ nanocrystals affords record efficiency of planar perovskite solar cells: Low‐temperature solution‐processed fluorinated TiO_2_ nanocrystals are applied as a novel electron transport layer of regular‐structure planar heterojunction perovskite solar cells, affording the best power conversion efficiency (PCE) of 22.68%. F‐doping improves the stabilities of devices, especially UV‐light stability due to inhibited photocatalytic activity of TiO_2_.

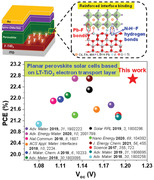

## 1 Introduction

During the past decade, tremendous attention has been paid to organic‐inorganic hybrid perovskite solar cells (PSCs) owing to the peculiar properties of organometal halide perovskite light‐absorbing materials, including tunable bandgaps, large absorption coefficients, high carrier mobilities, large carrier diffusion lengths, and small exciton binding energies.^[^ ^1^ ^]^ Thanks to the continuous efforts on engineering not only the composition, phase, and morphology of the perovskite absorber layer but also the perovskite/electrode interfaces, the record power conversion efficiency (PCE) of PSCs has reached 25.5%.^[^ ^2^, ^3^ ^]^ Even so, several critical issues such as device stability,^[^ ^4^ ^]^ scalability,^[^ ^5^ ^]^ and flexibility^[^ ^6^ ^]^ still hinder the commercialization road of PSCs.

Regular‐structure (n–i–p) planar heterojunction (PHJ) are the most commonly used architectures for high‐efficiency PSCs, in which an electron transport layer (ETL) sandwiched between the bottom transparent electrode and the perovskite layer atop plays an important role in efficient interfacial electron transport.^[^ ^7^ ^]^ Various metal oxides such as TiO_2_, SnO_2_, ZnO, and Nb_2_O_5_ have been used as ETLs in the PHJ‐PSCs, demonstrating different performances due to their difference on the electronic structures.^[^ ^8^ ^]^ Among them, low‐temperature (generally ≤150 °C) solution‐processed TiO_2_ nanocrystals are compatible with low‐cost fabrication and flexible devices, thus have been extensively applied.^[^ ^9^, ^10^, ^11^ ^]^ Nonetheless, the inherent drawbacks of low‐temperature solution‐processed TiO_2_ (LT‐TiO_2_), such as low electron mobility, high density of electronic trap states, and high photocatalytic activity, result in undesirable charge recombination at the ETL/perovskite interface and notorious instability of PSCs under ultraviolet (UV) light.^[^ ^12^, ^13^ ^]^ Such drawbacks of LT‐TiO_2_ can be overcome by either modulating its intrinsic electronic and band structures through elemental doping^[^ ^14^ ^]^ or tailoring its electric properties and morphology via surface modifications.^[^ ^7^ ^]^ In particular, despite of the success of elemental doping in modulating the intrinsic electronic and band structures of TiO_2_,^[^ ^10^, ^15^ ^]^ up to now doping LT‐TiO_2_ by nonmetallic elements for applications as ETL of PSCs has been scarcely reported.^[^ ^9^ ^]^ In 2016, Tan et al. developed a contact‐passivation strategy by capping LT‐TiO_2_ nanoparticles with chlorine (Cl), and found that Cl‐doping enhanced the contact of perovskite with TiO_2_ and suppressed interfacial recombination, leading to improved device stability along with an improved PCE of 20.9%.^[^ ^9^ ^]^ This limited report demonstrates that nonmetallic element doping of TiO_2_ is quite effective in not only modulating the intrinsic electronic and band structures of TiO_2_ itself but also promoting the quality of the perovskite layer atop. Nevertheless, it is unknown whether Cl‐doping can tackle the high photocatalytic activity drawback of TiO_2_ or not, thus the instability issue of TiO_2_‐based PSC devices under UV light remains unsolved. Hence, exploiting novel nonmetallic elements to dope LT‐TiO_2_ for not only improved ETL performance but also inhibited photocatalytic activity is highly desirable.

Herein, we develop a simple one‐step nonhydrolytic method to in situ surface fluorinate LT‐TiO_2_ nanocrystals, affording formation of Ti─F bonds and consequently increased electron mobility, decreased density of electronic trap states, and inhibited photocatalytic activity of TiO_2_ due to preferentially binding of fluorine (F) atom onto the (001) facet of anatase TiO_2_. Fluorinated TiO_2_ nanocrystals (abbreviated as F‐TiO_2_) is applied as a novel ETL in regular‐structure K_0.025_Cs_0.05_FA_0.83_MA_0.12_PbI_2.55_Br_0.45_ (abbreviated as KCsFAMA) PHJ‐PSC devices, delivering a champion PCE of 22.68%, which is among the highest PCEs for PHJ‐PSCs based on LT‐TiO_2_ ETLs. F‐doping not only reinforces interface binding of KCsFAMA perovskite layer with TiO_2_ ETL, but also contributes to effective passivation of the surface trap states of perovskite film as deduced from a series of characterizations and theoretical calculations. As a result, dramatic enhancements of device efficiency and stability especially under UV light are achieved.

## 2 Results and Discussion

Fluorine (F) has the highest electronegativity among all elements, and it has been reported that incorporation of F^−^ anion in perovskite layer led to passivation of both anion and cation vacancies of perovskite and consequently improved PSC device efficiency along with enhanced thermal and environmental stability.^[^ ^16^ ^]^ Besides, it has been reported that the photocatalytic activity of TiO_2_ may be inhibited by surface fluorine termination.^[^ ^17^, ^18^ ^]^ Hence, we manage to employ F as a dual‐functional dopant of TiO_2_ so as to investigate the feasibility of improving PSC efficiency and enhancing UV‐light stability in our present study.

**Figure 1:**
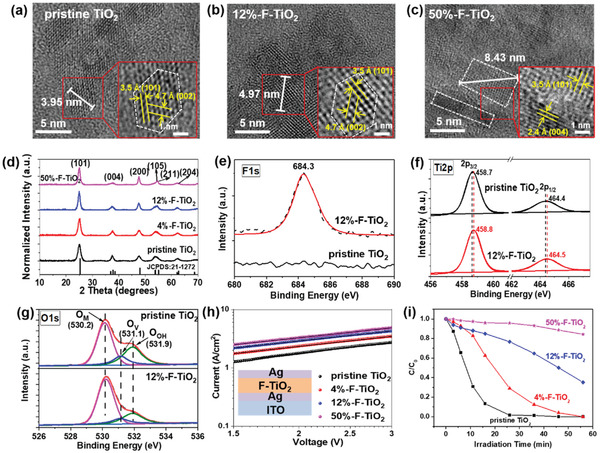
HR‐TEM images of the a) pristine TiO_2_, b) 12%‐F‐TiO_2_, and c) 50%‐F‐TiO_2_ nanocrystals dispersed in ethanol. d) XRD patterns of the pristine TiO_2_ and F‐TiO_2_ nanocrystals. e) The F 1s XPS spectra of the pristine TiO_2_ and 12%‐F‐TiO_2_ films. f) Ti 2p XPS spectra of pristine TiO_2_ and 12%‐F‐TiO_2_ films. g) The O 1s XPS spectra of the pristine TiO_2_ and 12%‐F‐TiO_2_ powders. h) Dark current–voltage curves of the electron‐only devices utilized for estimating the SCLC electron mobility. The inset is the corresponding structure of the electron‐only devices. i) Photocatalytic degradation of RhB over the different TiO_2_ powders under the simulated sunlight light. *C* and *C*_0_ denote the reaction and absorption equilibrium concentrations of RhB in the system.

In situ surface fluorination of TiO_2_ nanocrystals was fulfilled by a one‐step nonhydrolytic sol–gel method.^[^ ^11^ ^]^ Briefly, TiF_4_ was doped into a TiCl_4_ precursor solution dissolved in anhydrous ethanol with variable F:Ti molar ratios of 4%, 8%, 12%, 20%, and 50% (nominally abbreviated as 4%‐F‐TiO_2_, 8%‐F‐TiO_2_, 12%‐F‐TiO_2_, 20%‐F‐TiO_2_, and 50%‐F‐TiO_2_, respectively). F‐doped TiO_2_ (F‐TiO_2_) colloidal nanocrystals were obtained by dispersing the alcoholyzed TiCl_4_ in anhydrous benzyl alcohol in ethanol with titanium(diisopropoxide) bis(2,4‐pentanedionate) (TIPD) as the stabilizer (see the Experimental Section for details). Because of the much larger electronegativity of F than Cl, we found that TiF_4_ with stronger Ti─F ionic bonds is much more stable than TiCl_4_ during the alcoholysis process, thus Cl was completely removed by ligand exchange of TIPD and only F was doped in the final TiO_2_ colloidal nanocrystals. The optimized F:Ti molar ratio is determined to be 12% according to the device performance discussed below. To simplify the analyses, hereafter all characterizations are based on typical F:Ti molar ratios of 4% (low), 12% (optimal), and 50% (excess) unless otherwise stated. According to a comparison of the transmission electron microscopy (TEM) images of F‐TiO_2_ and the pristine TiO_2_ nanocrystals (Figure S1, Supporting Information), the obtained TiO_2_ nanocrystals are quasi‐monodispersed, and F‐doping leads to increase of the average particle size. This is confirmed by analyzing their corresponding high‐resolution (HR) TEM images (**Figure** **1**a–c), revealing that the lateral size of TiO_2_ nanocrystals increases from 3.95 nm (pristine TiO_2_) to 4.97 and 8.43 nm for 12%‐F‐TiO_2_ and 50%‐F‐TiO_2_, respectively. The lattice fringes of (101) plane with space distance of ≈3.5 Å are clearly observed for all samples, while those of (002) planes with space distance of 4.7 Å observed for the pristine TiO_2_ and 12%‐F‐TiO_2_ disappear for 50%‐F‐TiO_2_, which shows clearly lattice fringes of (004) plane instead. This indicates that F doping induces change of the exposed plane of TiO_2_ surface.

The effect of F‐doping on the crystalline phase of TiO_2_ is investigated by powder X‐ray diffraction (XRD). As shown in Figure 1d, the diffraction patterns of F‐TiO_2_ and the pristine TiO_2_ nanocrystals are in agreement with the standard pattern of crystalline anatase TiO_2_ (JCPDS:21‐1272). Interestingly, the full width at half‐maximum (FWHM) of the diffraction peaks (101) and (200) of F‐TiO_2_ decrease, while that of the diffraction peak (004) of F‐TiO_2_ increases relative to those of the pristine TiO_2_. This indicates that F‐doping leads to preferential growth of TiO_2_ crystal along (100) facet with the increase of the crystal size (200) facet estimated by Scherrer's equation (see Table S1 in the Supporting Information). Such a crystal shape change of TiO_2_ may be caused by the preferential binding of F atoms onto the (001) facets of anatase TiO_2_, resulting in reduced surface energy and kinetically inhibiting the crystal growth in the direction perpendicular to (001) facets, and consequently (001) facets are preferentially exposed.^[^ ^18^, ^19^ ^]^ Since the photocatalytic activity of TiO_2_ is sensitively dependent on the exposed crystalline facet and surface fluorine termination, F‐doping induced exposure of (001) facets is expected to weaken the photocatalytic activity of TiO_2_.^[^ ^17^, ^18^ ^]^

An intriguing question is whether F^−^ anion involved in TiF_4_ starting material exists as a dopant in the final TiO_2_ films. We carried out X‐ray photoelectron spectroscopic (XPS) characterizations to probe the change of chemical composition of TiO_2_ films before and after fluorination. As shown in the XPS survey spectra (Figure S2, Supporting Information), no F 1s signal is detected for the pristine TiO_2_ film, whereas F 1s signal appears obviously for 12%‐F‐TiO_2_ film, confirming the existence of F in the final TiO_2_ films. The valence state of F within F‐TiO_2_ is further studied by high‐resolution F 1s XPS spectroscopy, indicating a symmetrical peak centered at 684.3 eV (Figure 1e). This corresponds to F^−^ anion that binds onto the surface of TiO_2_ via Ti─F ionic bonds.^[^ ^18^, ^20^ ^]^ The actual atomic ratio of F:Ti is estimated to be 0.050 (see Table S2 in the Supporting Information). No any Cl signal is detected in both 12%‐F‐TiO_2_ and pristine TiO_2_ films, revealing no Cl contamination. Meanwhile, according to the Ti 2p XPS spectrum of 12%‐F‐TiO_2_, the Ti 2p_1/2_ and Ti 2p_3/2_ peaks with the binding energies of 464.5 and 458.7 eV shift positively by 0.1 eV relative to those of the pristine TiO_2_ (Figure 1f), agreeing with the formation of Ti─F ionic bonds due to the higher electronegativity of F than oxygen.^[^ ^21^ ^]^

It is known that oxygen vacancies as the major defects exist in LT‐TiO_2_ nanocrystals.^[^ ^22^, ^23^ ^]^ We investigated the influence of F‐doping on the oxygen vacancies by comparing the O 1s XPS spectra of the pristine TiO_2_ and 12%‐F‐TiO_2_ powders. As shown in Figure 1g, both the pristine TiO_2_ and 12%‐F‐TiO_2_ exhibit two asymmetric broad peaks, which can be deconvoluted into three peaks centered at ≈530.2, 531.1, and 531.9 eV, attributed to the O^2−^ anion bonded with the Ti^4+^ cation in the stoichiometric TiO_2_ structure (O_M_), the oxygen vacancies (O_V_), and the hydroxyl groups bonding to TiO_2_ (O_OH_), respectively.^[^ ^11^ ^]^ Interestingly, after fluorination, the relative content of oxygen vacancies (O_V_) within TiO_2_, which is estimated from the relative area of the O_V_ signal [O_V_/(O_M_ + O_V_ + O_OH_)], decreases from 15.6% to 12.3% (Table S3, Supporting Information). This indicates that F‐doping leads to passivation of the oxygen vacancies of TiO_2_.^[^ ^24^ ^]^ To understand the mechanism of F^−^ induced oxygen defect passivation of TiO_2_, we carry out density functional theory (DFT) calculations (see Note S4 in the Supporting Information for details). According to a comparison of the density of states (DOS) of the defective TiO_2_ before and after F incorporation, when only one F atom is doped within TiO_2_ unit, the incorporated F atoms tend to occupy the oxygen vacancies of TiO_2_, but have little influence on the defect energy level of the defective TiO_2_ (Figure S3e,f, Supporting Information). Surprisingly, with the increase of the number of doped F atoms from one to two, F may bond with the uncoordinated Ti in addition to occupying the oxygen vacancy, vanishing the defect energy level of TiO_2_ (Figure S3g,h, Supporting Information). However, upon increasing the number of doped F atoms to three, a new defect level appears near the valence band edge (Figure S3i,j, Supporting Information). The formation of Ti─F bonds on the surface of TiO_2_ may passivate oxygen vacancies and/or Ti dangling bonds on the surface of TiO_2_, thus efficiently suppressing electron–hole recombination. This is expected to enhance the electron mobility and decrease the photocatalytic activity of TiO_2_ as discussed below.^[^ ^19^, ^24^, ^25^, ^26^ ^]^

Since the chemical composition and crystalline lattice structure determine the optical and electronic properties of TiO_2_,^[^ ^25^, ^27^ ^]^ it is necessary to probe the influence of F‐doping on the optical and electronic properties of TiO_2_. The optical transmission spectra of the pristine TiO_2_ and F‐TiO_2_ films are compared in Figure S4 in the Supporting Information, revealing that F‐TiO_2_ films have higher optical transmittance in the range of 400–700 nm than the pristine TiO_2_ film likely due to Fresnel interference.^[^ ^28^ ^]^ Such an increase of in the optical transmittance allows more light to pass through the TiO_2_ ETL entering perovskite layer, beneficial for light absorbance of perovskite layer.^[^ ^6^ ^]^ On the other hand, F‐doping imposes negligible influence on the UV–vis optical absorption of TiO_2_, while the optical bandgap of TiO_2_ estimated from the diffuse reflectance spectra increases slightly from 3.13 eV (the pristine TiO_2_) to 3.17 eV (50%‐F‐TiO_2_) after F‐doping (Figure S5, Supporting Information), consistent with the passivated surface oxygen vacancies after F doping.^[^ ^12^ ^]^

The electron mobility of TiO_2_ film is measured by the space charge limited current (SCLC) method with a sandwiched structure of indium tin oxide (ITO)/Ag/TiO_2_/Ag.^[^ ^13^ ^]^ The corresponding *I*–*V* curves for the electron‐only devices based on different TiO_2_ films are illustrated in Figure 1h, from which the electron mobility (*µ*_e_) of the pristine TiO_2_, 4%‐F‐TiO_2_, 12%‐F‐TiO_2_, and 50%‐F‐TiO_2_ films are calculated to be 0.77 × 10^−4^, 1.21 × 10^−4^, 1.92 × 10^−4^, and 5.29 × 10^−4^ cm^2^ V^−1^ s^−1^, respectively (see Figure S6 and Table S4 in the Supporting Information). Clearly, the electron mobility increases dramatically by more than one order of magnitude for 50%‐F‐TiO_2_. This is interpreted by oxygen defects passivation and change of exposed crystalline plane after F doping.^[^ ^26^, ^29^ ^]^

To unravel the influence of F‐doping on the photocatalytic activity of TiO_2_, we evaluated the degradation rate of Rhodamine B (RhB) dye in the presence of TiO_2_ nanocrystals photocatalysts under irradiation of a solar simulator. Figure 1i compares the photocatalytic activities of the pristine TiO_2_, 4%‐F‐TiO_2_, 12%‐F‐TiO_2_, and 50%‐F‐TiO_2_, indicating that the photocatalytic activity of TiO_2_ decreases obviously after F‐doping, and the inhibition degree increases with the increase of the molar ratio of the incorporated F. However, after calcination at 600 °C in air for 3 h, the photocatalytic activity of 50%‐F‐TiO_2_ to degrade RhB was obviously higher than that of the pristine TiO_2_ (Figure S7, Supporting Information). It has been reported that the photocatalytic activity of TiO_2_ is primarily determined by the oxygen vacancy and Ti unsaturated site^.[^ ^17^ ^]^ Hence, the inhibited photocatalytic activity of TiO_2_ after F‐doping is understandable since F‐doping leads to passivation of the oxygen vacancy and Ti dangling bonds as discussed above.

We next employed scanning electron microscopy (SEM) and atomic force microscopy (AFM) to probe the influence of F‐doping on the morphology of TiO_2_ film, which is essential for the deposition of perovskite layer atop. Figure S8a–d in the Supporting Information compare the SEM images of the pristine TiO_2_ and F‐TiO_2_ films deposited on ITO substrate, indicating that their overall film morphologies are similar. According to AFM measurements, the film smoothness of 12%‐F‐TiO_2_ is also comparable to that of the pristine TiO_2_ as evidenced from their comparable root‐mean‐square (RMS) roughness, while increasing further the F content for 50%‐F‐TiO_2_ results in obvious increase of RMS roughness from 4.6 to 11.0 nm (Figure S8e–h, Supporting Information). Indeed, 50%‐F‐TiO_2_ film looks less uniform than the pristine TiO_2_ and 12%‐F‐TiO_2_, composed of random aggregations and many pinholes. Such a rough and nonuniform film is likely due to the increase of lateral size of TiO_2_ nanocrystals and expected to deteriorate the interfacial contact between TiO_2_ ETL and perovskite, consequently detrimental for interfacial electron transport as discussed below.^[^ ^30^ ^]^

**Figure 2:**
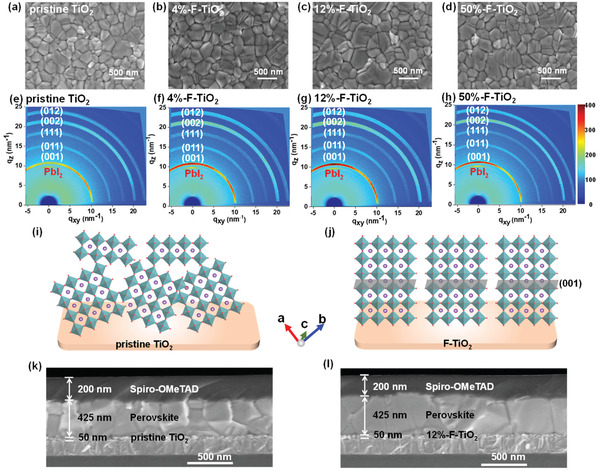
Surface SEM images and 2D‐GIXRD profiles of the perovskite films deposited on different TiO_2_ ETLs: a,e) pristine TiO_2_, b,f) 4%‐F‐TiO_2_, c,g) 12%‐F‐TiO_2_, and d,h) 50%‐F‐TiO_2_. Schematic illustration of 3D perovskite crystals deposited on the i) pristine TiO_2_ and j) 12%‐F‐TiO_2_ substrates. Cross‐sectional SEM images of perovskite films deposited on the k) pristine TiO_2_ and l) 12%‐F‐TiO_2_ substrates.

It is then necessary to examine whether such morphological changes of TiO_2_ film induced by F‐doping affect the KCsFAMA perovskite films atop. We deposited KCsFAMA perovskite films under identical conditions onto different TiO_2_ substrates, and examined the morphology and crystallinity of the perovskite film by using AFM, SEM, and synchrotron‐based grazing incidence X‐ray diffraction (GIXRD). As illustrated from the SEM images of the perovskite films deposited on different TiO_2_ substrates (**Figure** **2**a–d), all perovskite films appear uniform and dense without discernible pinholes, while an obvious increase of the average crystalline grain size from ≈390 nm (the pristine TiO_2_) to ≈410 nm (4%‐F‐TiO_2_) and ≈470 nm (12%‐F‐TiO_2_) is observed. Intriguingly, upon increasing further the F content for 50%‐F‐TiO_2_, the average crystalline grain size decreases to ≈460 nm (Figure S9, Supporting Information). This reveals that F‐doping may affect the crystal nucleation and crystallization kinetics of perovskite.^[^ ^16^ ^]^ In addition, compared with the perovskite film deposited on the pristine TiO_2_ substrate, the perovskites films deposited on F‐TiO_2_ substrates become smoother as deduced from their smaller RMS roughnesses (Figure S10, Supporting Information).

Figure 2e–h compares the 2D‐GIXRD profiles of the KCsFAMA perovskite films deposited on different TiO_2_ substrates obtained under an X‐ray incident angle of 0.2°. The diffraction profiles of all perovskite films are similar, exhibiting several characteristic scattered rings at *q* ≈ 10, 20, and 22.2 nm^−1^ corresponding respectively to the (001), (002), and (012) crystal planes of perovskite. This feature is consistent with those reported in the literature.^[^ ^31^ ^]^ After F‐doping, all of the scattered rings of perovskite films look brighter than those of perovskite deposited on the pristine TiO_2_, indicating that F‐doping of TiO_2_ leads to improved crystallinity of perovskite film atop. This statement is verified more intuitively by comparing the radially integrated intensity of the rings at *q* between 5 and 30 nm^−1^ (Figure S11a, Supporting Information). Clearly, the (001), (002), and (012) peak intensities increase for the perovskite films deposited on the F‐TiO_2_ substrates relative to those for the perovskite on the pristine TiO_2_. Besides, crystalline orientation of perovskite films deposited on different TiO_2_ substrates are compared in the azimuthal scan profiles along the ring of (001) plane extracted from the 2D‐GIXRD patterns (Figure S11b, Supporting Information). Unlike the perovskite film on the pristine TiO_2_ substrate which shows the lowest intensity at 90°, the peaks at 90° for the perovskite films on F‐TiO_2_ substrates become the most intense, suggesting that F‐doping of TiO_2_ affords preferential out‐of‐plane orientation of (001) plane for perovskite film (Figure 2i,j).^[^ ^31^ ^]^ This conclusion is solidified by comparing the cross‐sectional SEM images of the perovskite films deposited on different TiO_2_ substrates, revealing that the crystal grains of perovskite film (≈425 nm thick) deposited on 12%‐F‐TiO_2_ (≈50 nm thick) are columnar with vertical orientations, whereas the control perovskite film deposited on the pristine TiO_2_ exhibits random orientation of crystalline grains (Figure 2k,l). Such oriented growth of perovskite crystal grain is expected to promote the vertical charge transport within the perovskite layer.^[^ ^31^, ^32^ ^]^

To understand the origin of F‐induced oriented growth of perovskite crystal grains, we carried out a series of characterizations to probe the interaction of F‐TiO_2_ with KCsFAMA perovskite. We first spin‐coated a 50%‐F‐TiO_2_ nanocrystals suspension dispersed in isopropanol onto KCsFAMA perovskite film, and used XPS to study the interactions of F‐TiO_2_ with KCsFAMA perovskite. As shown in the high‐resolution Pb 4f XPS spectra, for the perovskite/F‐TiO_2_ film the Pb 4f_5/2_ and Pb 4f_7/2_ signals detected at 142.9 and 138.0 eV both shift positively by 0.1 eV relative to those detected for the pristine TiO_2_ (Figure S12a, Supporting Information), suggesting the formation of Pb─F ionic bonds at perovskite/F‐TiO_2_ interface.^[^ ^33^ ^]^ The Pb─F bond is expected to suppress Pb–I antisite defects at the perovskite/TiO_2_ interface, as discussed further below.^[^ ^9^ ^]^ Besides, N 1s XPS signal also shifts positively for the perovskite/F‐TiO_2_ film (Figure S12b, Supporting Information). Given the strong electronegativity of fluorine, such a shift of N 1s peak suggests the change of N─H bonding within MA/FA cations, which is resulted from the formation of hydrogen bonds (N─H···F) between fluorine and MA/FA cations.^[^ ^16^ ^]^ To verify this, we carried out solid‐state ^19^F NMR measurements of a mixture of 50%‐F‐TiO_2_ and formamidinium iodide (FAI), and find that the ^19^F signal of the original 50%‐F‐TiO_2_ shifts toward downfield after mixed with FAI (Figure S12c, Supporting Information), confirming the formation of N─H···F hydrogen bonds between fluorine and MA/FA cations.^[^ ^34^ ^]^ As a result, the doped F atom bridges TiO_2_ and perovskite via the Pb─F ionic bonds and N─H···F hydrogen bonds are expected to reinforce the interface binding of perovskite layer with TiO_2,_ benefiting the oriented growth of perovskite crystal grains (Figure S12d,e, Supporting Information).^[^ ^16^ ^]^

**Figure 3:**
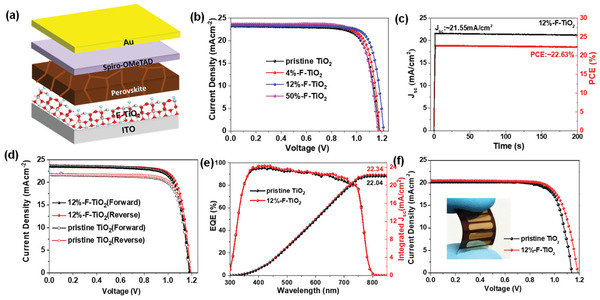
a) Schematic structure of ITO/F‐TiO_2_/KCsFAMA perovskite/spiro‐OMeTAD/Au device. b) *J*–*V* curves of the champion devices based on different TiO_2_ ETLs. The scanning direction is from open‐circuit voltage to short circuit (reverse) with a scan rate of 0.1 V s^−1^. c) The stabilized photocurrent and power output of the PSC devices based on 12%‐F‐TiO_2_ ETL measured at the maximum power points (1.05 V). d) *J*–*V* curves of the devices based on pristine TiO_2_ and 12%‐F‐TiO_2_ ETLs under reverse and forward scans with a scan rate of 0.1 V s^−1^. e) EQE spectra of the devices based on pristine TiO_2_ and 12%‐F‐TiO_2_ ETLs. f) *J*–*V* curves of the champion flexible devices based on pristine TiO_2_ and 12%‐F‐TiO_2_ ETLs. The scanning direction is from open‐circuit voltage to short circuit (reverse) with a scan rate of 0.1 V s^−1^. The inset shows the photograph of a flexible device.

Given that F‐doping not only leads to passivation of the oxygen vacancy and increase of electron mobility of TiO_2_, but also induces oriented growth of KCsFAMA perovskite crystal grains, it is necessary to investigate whether such effects benefit PSC device performance or not. We incorporated F‐TiO_2_ nanocrystals as novel ETLs and fabricated the regular‐structure (n–i–p) PHJ‐PSC devices with the structure of ITO/F‐TiO_2_/KCsFAMA perovskite/2,2′,7,7′‐tetrakis(*N*,*N*‐di‐*p*‐methoxyphenylamine)‐9,9‐spirobifluorene (spiro‐OMeTAD)/Au (**Figure** **3**a). Figure 3b compares the current density–voltage (*J*–*V*) curves of the champion devices based on different TiO_2_ ETLs measured under irradiation of 100 mW cm^−2^ (AM 1.5G). The corresponding photovoltaic parameters, including open‐circuit voltage (*V*_oc_), short‐circuit current (*J*_sc_), fill factor (FF), PCE, series resistance (*R*_s_), and shunt resistance (*R*_sh_), obtained on the basis of the statistical data of over 60 devices fabricated independently are summarized in Table 1 (see also Figure S13 and Table S5 in the Supporting Information for the photovoltaic data obtained from F‐TiO_2_ ETLs with other F:Ti molar ratios). The control device based on the pristine TiO_2_ ETL exhibits a best PCE of 21.09% calculated from a *V*_oc_ of 1.17 V, a *J*_sc_ of 23.23 mA cm^−2^, and an FF of 77.33%. Upon using F‐TiO_2_ ETL, both the average and best PCEs increase obviously, and the device based on 12%‐F‐TiO_2_ ETL delivers the highest champion PCE of 22.68%, derived from a *V*_oc_ of 1.21 V, a *J*_sc_ of 23.36 mA cm^−2^, and an FF of 80.25%. Thus, the optimized F:Ti molar ratio is determined to be 12%. Besides, the stabilized photocurrent and steady PCE for the champion 12%‐F‐TiO_2_ device loaded at the maximum power point (*V*_mmp_ ≈ 1.05 V) over 200 s reach 21.55 mA cm^−2^ and 22.63%, respectively, significantly higher than those of the control device (Figure 3c; Figure S14, Supporting Information). Moreover, both 12%‐F‐TiO_2_ and pristine TiO_2_ devices exhibit negligible hysteresis, while the hysteresis index defined as [PCE(reverse) − PCE(forward)]/PCE(reverse)^[^ ^35^ ^]^ decreases slightly for 12%‐F‐TiO_2_ device (Figure 3d; Table S6, Supporting Information). Remarkably, to our knowledge the record PCE for the PHJ‐PSC devices based on low‐temperature solution‐processed TiO_2_ ETLs reported up to now is 22.7%,^[^ ^36^ ^]^ hence the highest PCE of 22.68% obtained in our present work is among the highest PCEs for devices based on LT‐TiO_2_ ETLs (see Table S7 and Figure S15 in the Supporting Information).

**Table 1:** Photovoltaic parameters of the perovskite solar cells deposited on different TiO_2_ ETLs under one sun illumination (AM 1.5G, 100 mA cm^−2^)

ETL	Molar ratio of F:Ti [%]^a)^		*V*_oc_[V]	*J*_sc_ [mA cm^−2^]	FF[%]	PCE[%]	*R*_s_ ^b)^[Ω cm^2^]	*R*_sh_ ^b)^[Ω cm^2^]
Pristine TiO_2_	0	Average^c)^	1.16 ± 0.02	22.35 ± 0.79	73.86 ± 2.82	19.17 ± 1.11	4.58	2272.36
		Champion	1.17	23.23	77.33	21.09	3.79	3578.55
4%‐F‐TiO_2_	4	Average^c)^	1.16 ± 0.02	22.74 ± 0.74	75.11 ± 2.20	19.87 ± 1.05	4.28	2919.47
		Champion	1.18	23.47	78.69	21.80	3.25	3433.31
12%‐F‐TiO_2_	12	Average^c)^	1.17 ± 0.02	22.90 ± 0.61	77.03 ± 1.90	20.66 ± 0.97	3.89	3706.49
		Champion	1.21	23.36	80.25	22.68	3.32	8534.90
50%‐F‐TiO_2_	50	Average^c)^	1.15 ± 0.03	22.96 ± 0.68	74.89 ± 2.38	19.79 ± 0.97	4.08	2740.63
		Champion	1.16	23.71	78.05	21.55	3.95	9206.54

^a)^ The molar ratio of F^−^ relative to Ti^4+^ in the raw solution

^b)^ *R*_s_ and *R*_sh_ are given by the PCE measurement system

^c)^ Averaged over 60 devices fabricated independently.

We next compared the three key photovoltaic parameters (*V*_oc_, *J*_sc_, and FF) of 12%‐F‐TiO_2_ and pristine TiO_2_ devices based on the statistic data of over 60 independent devices, and find that *V*_oc_, *J*_sc_, and FF all increase for 12%‐F‐TiO_2_ device, while the PCE enhancement from 19.17% to 20.66% (≈7.8% enhancement) is primarily resulted from increases of both *J*_sc_ (from 22.35 to 22.90 mA cm^−2^, ≈2.5% enhancement) and FF (from 73.86% to 77.03%, ≈4.3% enhancement) (see Table 1 and Figure S16 in the Supporting Information). The increase of *J*_sc_ is corroborated by external quantum efficiency (EQE) measurements, indicating that the overall EQE responses of 12%‐F‐TiO_2_ devices are slightly higher than that of the control pristine TiO_2_ device (Figure 3e), consequently a larger integrated *J*_sc_ is obtained. The superior ETL performance of F‐TiO_2_ along with its low‐temperature solution‐processibility stimulates us to evaluate its suitability for flexible PSC devices. Similar to rigid ITO‐based devices, flexible PSCs based on polyethylene naphthalate (PEN) substrates were fabricated using the same process. The *J*–*V* curves of the champion PEN/ITO/F‐TiO_2_/KCsFAMA perovskite/spiro‐OMeTAD/Au flexible PSC devices based on the pristine TiO_2_ and F‐TiO_2_ ETLs measured under irradiation of 100 mW cm^−2^ (AM 1.5G) are compared in Figure 3f, and the corresponding statistical photovoltaic parameters of over 20 devices are summarized in Table S8 in the Supporting Information. Similar to the case of ITO‐based devices, all flexible PSC devices based on F‐TiO_2_ ETLs show higher average and champion PCEs, and 12%‐F‐TiO_2_ devices deliver the highest values. This result reveals that F‐doping strategy is universal for both rigid and flexible substrates. In particular, the highest PCE of 18.26% obtained for 12%‐F‐TiO_2_ flexible PSC device appears to the record value for flexible PHJ‐PSC based on LT‐TiO_2_ ETLs (Table S9 and Figure S17 in the Supporting Information).

To understand the change of the *V*_oc_ value after F‐doping, the energy level alignment between the TiO_2_ ETL and perovskite needs to be checked. We used ultraviolet photoelectron spectroscopy (UPS) to probe the effect of F‐doping on the work function of TiO_2_. The determined work function (*W*_F_) of the pristine TiO_2_, 4%‐F‐TiO_2_, 12%‐F‐TiO_2_, and 50%‐F‐TiO_2_ are 4.10, 4.15, 4.26, and 4.31 eV, respectively (see Figure S18 in the Supporting Information), revealing that F‐doping leads to the increase of the work function of TiO_2_. Such a downshift of the Fermi level of TiO_2_ would result in the decrease of built‐in potential (*V*_bi_) in the device, consequently leading to the decrease of *V*_oc_ for 50%‐F‐TiO_2_ device.^[^ ^37^ ^]^

**Figure 4:**
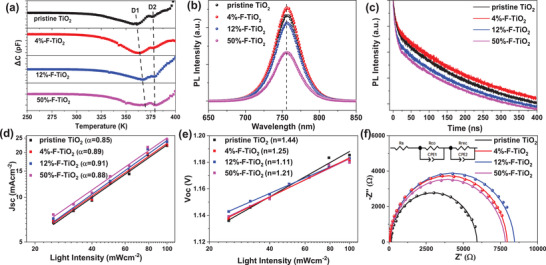
a) DLTS spectra of the devices based on different TiO_2_ ETLs. b) Steady‐state PL and c) TRPL spectra of perovskite films deposited on different TiO_2_ substrates. Responses of d) *J*_sc_ and e) *V*_oc_ on the irradiation intensity of the devices based on different TiO_2_ ETLs. f) Nyquist plots of the devices based on different TiO_2_ ETLs measured in the dark under a reverse potential of 1.0 V. The fitted curves are shown as solid lines, and the experimental data are shown as corresponding points. Inset: The equivalent circuit model employed for the fitting of the impedance spectra.

It is known that the existence of defects of the perovskite, which mainly distribute on the surface of polycrystalline film, is an intrinsic factor deteriorating the device performance.^[^ ^38^ ^]^ We investigated the effect of F‐doping on defect passivation of perovskite film by measuring the electron trap‐state density using SCLC method based on an electron‐only device with a structure of ITO/F‐TiO_2_/KCsFAMA perovskite/PCBM/Ag.^[^ ^39^ ^]^ According to a comparison of the *I*–*V* curves of the devices based on different TiO_2_ ETLs (Figure S19, Supporting Information), the electron trap‐state density (*n*_t_) within the KCsFAMA perovskite films deposited on 4%‐F‐TiO_2_, 12%‐F‐TiO_2_, 50%‐F‐TiO_2_, and the pristine TiO_2_ substrates are calculated to be 5.90 × 10^15^, 3.59 × 10^15^, 9.49 × 10^15^, and 6.67 × 10^15^ cm^−3^, respectively (see Table S10 in the Supporting Information for detailed analyses). Hence, 12%‐F‐TiO_2_ device exhibits the lowest electron trap‐state density, which is decreased by around 46% relative to that for the control device. This is understandable since it has been reported that introduction of a moderate amount of F suppressed the FA vacancies defect at the surface of KCsFAMA perovskite through forming hydrogen bonds (N─H···F).^[^ ^16^ ^]^ However, excess F‐doping in the case of 50%‐F‐TiO_2_ results in increase of the electron trap‐state density from 6.67 × 10^15^ to 9.49 × 10^15^ cm^−3^. To gain deeper insight into the mechanism of F‐induced defect passivation of perovskite, we quantitatively measured the deep traps of perovskite by the deep‐level transient spectroscopy (DLTS). **Figure** **4**a compares the DLTS spectra of the devices based on different TiO_2_ ETLs.^[^ ^40^ ^]^ The activation energy (*E*_T_ − *E*_V_ or *E*_C_ − *E*_T_) and the concentration (*N*_T_) of traps can be obtained from Arrhenius plots and the fitting results (see Figure S20 and Table S11 in Note S23 in the Supporting Information for details). Two negative peaks are observed for all devices, which are assigned to hole traps (H) in n‐type perovskite films, labelled as D_1_ and D_2_, respectively.^[^ ^41^ ^]^ According to the activation energy of the deep traps, D_1_ and D_2_ could be attributed to antisite defects (such as I_Pb_ (or F_Pb_) and Pb_I_).^[^ ^42^ ^]^ After F doping, the D_1_ peaks shift to higher temperatures, suggesting the higher activation energies of the F_Pb_ than that of I_Pb_ defects (see Note S24 in the Supporting Information for details).The trap‐state densities of D_1_ defect within perovskite films based on different TiO_2_ ETLs decrease from 9.72 × 10^14^ (the pristine TiO_2_) to 6.69 × 10^14^ (4%‐F‐TiO_2_), 4.75 × 10^14^ (12%‐F‐TiO_2_), and 3.82 × 10^14^ (50%‐F‐TiO_2_) (see Figure S20 and Table S11 in Note S23 in the Supporting Information for details). The decreased D_1_ trap‐state density is resulted from the higher formation energy of F_Pb_ defect (3.022 eV) than that of I_Pb_ defect (2.869 eV) as predicted by DFT calculations (see Table S12 in the Supporting Information for details). In addition, the trap‐state densities of D_2_ defect associated with Pb_I_ are calculated to be 4.01 × 10^14^, 2.20 × 10^14^, 6.30 × 10^14^, and 5.71 × 10^14^ cm^−3^ within the perovskite films deposited on 4%‐F‐TiO_2_, 12%‐F‐TiO_2_, 50%‐F‐TiO_2_ and the pristine TiO_2_ substrates, respectively. Hence, partial substitution of I^−^ on the surface of perovskite by F^−^ can effectively suppress the Pb_I_ antisite defect. On the contrary, the 50%‐F‐TiO_2_ device exhibits the increased concentrations of D_2_ defect peaks relative to the 12%‐F‐TiO_2_ device, indicating that excess F doping leads to increased Pb_I_ antisite defects. It is known that deep‐level defects are the predominant trap sources for nonradiative recombination losses, resulting in deficit of *V*_oc_.^[^ ^43^ ^]^ Therefore, the suppressed deep‐level antisite defects after F‐doping explains the slight increase in *V*_oc_ value.

In order to unveil the factors responsible for the increases of *J*_sc_ and FF, we implemented a series of characterizations to elucidate the influence of F‐doping on the charge carrier dynamics of the device. It has been reported that the interfacial charge extraction sensitively determines *J*_sc_.^[^ ^44^ ^]^ The electron extraction and electron–hole recombination dynamics at the TiO_2_/perovskite interface is first investigated by steady‐state and time‐resolved photoluminescence (TRPL) spectroscopies. Figure 4b compares the steady‐state PL spectra of the KCsFAMA perovskite films deposited on different TiO_2_ substrates measured under an excitation wavelength of 460 nm, indicating that the characteristic PL peak at 756 nm of KCsFAMA perovskite film deposited on the pristine TiO_2_ substrate is quenched for those on 12%‐F‐TiO_2_ and 50%‐F‐TiO_2_, whereas such a PL intensity increases for the case of 4%‐F‐TiO_2_. Since the PL intensity of perovskite film is a balance of charge transfer and nonradiative recombination at interfaces,^[^ ^45^ ^]^ the increase of PL intensity for 4%‐F‐TiO_2_ may result from the suppression of nonradiative recombination by the passivation of perovskite defects.^[^ ^44^ ^]^ Upon increasing the F‐doping ratio, the quenching of PL peak for 12%‐F‐TiO_2_ and 50%‐F‐TiO_2_ is resulted from the more dramatic increases of the electron mobility of TiO_2_ as discussed above, leading to more efficient electron transfer from the KCsFAMA perovskite to TiO_2_ ETL, consequently contributing to increase of *J*_sc_. This conclusion is confirmed by the result of TRPL, revealing that the carrier lifetimes (*τ*) of the KCsFAMA perovskite films deposited on 12%‐F‐TiO_2_ and 50%‐F‐TiO_2_ (246.87 and 219.51 ns, respectively) are smaller than that on the pristine TiO_2_ (252.92 ns; Figure 4c; Table S13, Supporting Information).

We next investigate the dependence of *J*_sc_ and *V*_oc_ on light intensities (*I*) so as to understand further the recombination kinetics within the device.^[^ ^40^, ^47^ ^]^ The bimolecular recombination kinetics of the devices are probed by the ideality factors (*α*) extracted from the equation *J*_sc_∝*I^*α*^*, and the corresponding fitting curves are plotted in Figure 4d. The *α* values of devices based on the pristine TiO_2_, 4%‐F‐TiO_2_, 12%‐F‐TiO_2_, and 50%‐F‐TiO_2_ are 0.85, 0.89, 0.91, and 0.88, respectively. The largest *α* value of the 12%‐F‐TiO_2_ device, which is more close to unity, indicates the least bimolecular recombination.^[^ ^40^ ^]^ Besides, the dependence of voltage to the light intensity is characterized so as to reveal the trap‐assisted Shockley–Read–Hall (SRH) monomolecular recombination of device according to the equation *V*_oc_ = *nkT*ln(*I*)/*e* + constant (where *n* is the ideality factor, *k* is the Boltzmann constant, *T* is the absolute temperature, and *e* is the elementary charge).^[^ ^47^ ^]^ The dependence of *V*_oc_ on the logarithm of the incident light intensity are shown in Figure 4e, from which the extracted *n* values of devices based on the pristine TiO_2_, 4%‐F‐TiO_2_, 12%‐F‐TiO_2_, and 50%‐F‐TiO_2_ are determined to be 1.44, 1.25, 1.11, and 1.21, respectively. The *n* value of the 12%‐F‐TiO_2_ device is the closest to unity, suggesting the most effective suppression of the monomolecular SRH recombination. The largest suppression of both bimolecular and monomolecular SRH recombinations for the 12%‐F‐TiO_2_ devices is due to the passivation of antisite defects of perovskite and increased electron mobility of TiO_2_ as discussed above, which contribute directly to the increase in FF.^[^ ^11^, ^46^ ^]^

The interfacial charge transport properties of the PSC devices based on different TiO_2_ ETLs are probed by the electrochemical impedance spectroscopy (EIS) measured in the dark under a reverse potential of 1.0 V.^[^ ^48^ ^]^ Figure 4f presents the Nyquist plots of devices based on different TiO_2_ ETLs and the corresponding fitted curves obtained on the basis of a commonly used equivalent circuit model (see the inset in Figure 4f). The fitted parameters of *R*_co_ (contact resistance) and *R*_rec_ (recombination resistance) corresponding respectively to the electron transport and the electron–hole recombination are listed in Table S14 in the Supporting Information .^[^ ^11^ ^]^ Compared to the control device, the *R*_co_ values of F‐TiO_2_ devices decrease along with the increase of *R*_rec_ values, revealing that F‐doping enhances the electron transport and suppresses the electron–hole recombination at TiO_2_/perovskite interface. In this sense, the smallest *R*_co_ value and largest *R*_rec_ value of 12%‐F‐TiO_2_ device indicate the most efficient interfacial electron transport, contributing directly to the increase of *J*_sc_.^[^ ^13^ ^]^

**Figure 5:**
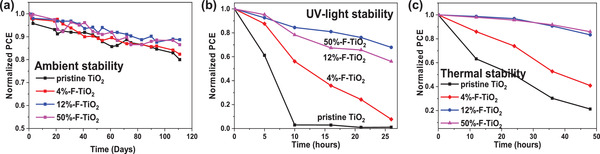
a) Ambient stabilities of the devices based on different TiO_2_ ETLs by storing the unencapsulated device in ambient condition (temperature: 25 °C; relative humidity: 35%) for 110 days. b) UV‐light stabilities of the encapsulated devices based on different TiO_2_ ETLs under high‐voltage mercury lamp irradiation (500 W) with 365 nm cutoff filter (temperature: 30 °C; relative humidity: 80%) for 26 h. c) Thermal stabilities of the devices based on different TiO_2_ ETLs by storing the unencapsulated device at 85 °C in a glove box for 48 h.

In the end, we examined the effect of F‐doping on the ambient, UV‐light, and thermal stability of the PSC devices by storing the devices under different conditions. First, the storage lifetime of the unencapsulated devices was monitored in ambient condition (temperature: 25 °C; relative humidity: 35%) for over 110 days. Compared to the control device based on the pristine TiO_2_ ETL for which PCE drops to ≈80% of the initial value due to the decomposition of the KCsMAFA perovskite,^[^ ^49^ ^]^ the 12%‐F‐TiO_2_ device exhibits improved stability with PCE retaining 88% after 110 days (**Figure** **5**a; Figure S22, Supporting Information). This indicates improved resistance to the attack of the moisture for 12%‐F‐TiO_2_ device owing to the improved quality and passivated detects of the perovskite film as discussed above.^[^ ^9^, ^50^ ^]^ Besides, the UV‐light stability of the devices encapsulated with ethoxyline was also evaluated under high‐voltage mercury lamp irradiation (500 W) with a 365 nm cutoff filter (temperature: 30 °C; relative humidity: 80%). As shown in Figure 5b, the 12%‐F‐TiO_2_ device retains 68% of the initial PCE after 26 h continuous UV light irradiation, whereas PCE of the control device degrades to nearly zero after only 10 h UV light irradiation. This reveals that F‐doping significantly increase the UV‐light stability of the device. Such an increase of UV‐light stability should result from the inhibited photocatalytic activity of TiO_2_ after F‐doping as discussed above. Moreover, the thermal stability of the unencapsulated device was examined by aging them under continuous heating at 85 °C in a dark nitrogen atmosphere in a glove box. The PCE of the 12%‐F‐TiO_2_ device decays very slowly and the PCE retains 83% of the initial value after 48 h, while the PCE of the control devices drops to ≈20% only (Figure 5c). This reveals that F‐doping improves the thermal stability of devices as well.

## 3 Conclusion

In summary, LT‐TiO_2_ nanocrystals were in situ fluorinated via a simple one‐step nonhydrolytic method, and the fluorinated TiO_2_ nanocrystals (F‐TiO_2_) were applied as a novel ETL in regular‐structure KCsFAMA PHJ‐PSC devices, affording a champion PCE of 22.68%, which is among the highest PCEs for devices based on LT‐TiO_2_ ETLs. F‐doping leads to formation of Ti─F bonds and consequently increased electron mobility, decreased density of electronic trap states and inhibited photocatalytic activity of TiO_2_ due to preferentially binding of fluorine (F) atom onto the (001) facet of anatase TiO_2_. Furthermore, F‐doping not only reinforces interface binding of KCsFAMA perovskite layer with TiO_2_ ETL through formation of Pb─F ionic bonds and N─H···F hydrogen bonds between F and MA/FA cations, but also contributes to effective passivation of the surface trap states of perovskite film. As a result, in addition to efficiency enhancement, F‐doping leads to improvements of the ambient and thermal stabilities of PSC devices, especially UV‐light stability due to inhibited photocatalytic activity of TiO_2_. With these findings, our study paves the way for low‐temperature fabrication of high‐efficiency perovskite solar cells.

## 4 Experimental Section

##### Materials

The ITO glass substrate with a sheet resistance of 10 Ω sq^−1^ was purchased from Shenzhen Nan Bo Group, China. All the chemicals were used as received, including FAI (Dyesol), methylammonium bromide (MABr, Dyesol), lead iodide (PbI_2_, 99.999%, TCI), lead bromide (PbBr_2_, 99.999%, Alfa Aesar), KI and CsI (MAI, Xi'an Polymer Light Technology Corp), TiCl_4_ (GR, 99.5%, Aladdin), TiF_4_ (Alfa Aesar), TIPD (TCI), spiro‐OMeTAD (1 M company), and Li‐bis(trifluoromethanesulfonyl) imide (Li‐TFSI, Sigma). Dimethylformamide (DMF), benzyl alcohol, chlorobenzene, and dimethyl sulfoxide (DMSO) were purchased from Sigma‐Aldrich. All materials were used as received without further purification.

##### Syntheses of F‐TiO_2_ Nanocrystals

The pristine TiO_2_ nanocrystals were synthesized by using a modified nonhydrolytic sol–gel method with all procedures done in the ambient condition.^[^ ^4^, ^8^ ^]^ First, 0.5 mL TiCl_4_ was injected drop by drop into 2 mL cold anhydrous ethanol with strong stirring. After the solution cooled down to room temperature, 10 mL of anhydrous benzyl alcohol was added to the previous solution and stirred for 10 min. The mixed solution was firmly sealed and stored without stirring in an oil bath pan at 85 °C for 12 h. The product of TiO_2_ nanocrystals was then precipitated from the as‐obtained solution by the addition of diethyl ether and isolated by centrifugation at 5000 rpm for 2 min. The solid was subsequently washed by adding anhydrous ethanol and diethyl ether, followed by a similar centrifugation step (5000 rpm for 2 min) for three times.

The F‐TiO_2_ nanocrystals were obtained with a similar approach except that TiF_4_ was doped into a TiCl_4_ solution dissolved in anhydrous ethanol as a precursor with variable F:Ti molar ratios of 4%, 8%, 12%, 20%, and 50% (nominally abbreviated as 4%‐F‐TiO_2_, 8%‐F‐TiO_2_, 12%‐F‐TiO_2_, 20%‐F‐TiO_2_, and 50%‐F‐TiO_2_, respectively). To stabilize the as‐obtained TiO_2_ and F‐TiO_2_ nanocrystals, the washed nanocrystals were dispersed in anhydrous ethanol (concentration of around 5 mg mL^−1^) by the addition of TIPD (15 µL mL^−1^).

##### Device Fabrication

A patterned ITO glass substrate was cleaned by sequential ultrasonic treatment in detergent, deionized water, acetone, and isopropyl alcohol. The as‐prepared TiO_2_ nanocrystals were spin‐coated onto the ITO substrate at 5000 rpm for 40 s twice, and then these samples were annealed at 150 °C for 25 min in air under ambient pressure. The following coating steps were performed under argon atmosphere inside a glovebox. For the fabrication of K_0.025_Cs_0.05_(FA_0.87_MA_0.13_)_0.95_PbI_2.55_Br_0.45_ (KCsFAMA) perovskite, the KCsFAMA precursor solution (1.3 m) was prepared with molar ratios of PbI_2_/PbBr_2_ = 1.1:0.2, FAI/MABr = 1:0.2, KsI/CsI/(FAI+MABr) = 0.025:0.05:0.95, PbI_2_/FAI = 1.1:1, and PbBr_2_/MABr = 1:0.2. The perovskite films were deposited onto the TiO_2_ substrates with two‐step spin coating procedures. The first step was 2000 rpm for 10 s with an acceleration of 200 rpm s^−1^. The second step was 6000 rpm for 30 s with a ramp‐up of 2000 rpm s^−1^. Chlorobenzene (100 µL) was dropped on the spinning substrate during the second spin‐coating step at 15 s before the end of the procedure. The substrate was then immediately transferred on a hotplate and heated at 100 °C for 60 min, followed by cooling down to room temperature naturally.

For the fabrication of KCsFAMA‐based flexible perovskite solar cells, the process and materials used were exactly the same as the rigid perovskite solar cells, except that the PEN/ITO (Peccell, Japan) instead of ITO glass was used as the substrate.

##### Measurements and Characterization

The current density–voltage (*J*–*V*) characterizations were measured by a Keithley 2400 source measurement unit under simulated AM 1.5 irradiation (100 mW cm^−2^) with a standard xenon‐lamp‐based solar simulator (Oriel Sol 3A, USA), which was calibrated with a monocrystalline silicon reference cell (Oriel P/N 91150 V, with KG‐5 visible color filter) calibrated by the National Renewable Energy Laboratory (NREL). Around 60 devices were fabricated and measured independently under each experimental condition to confirm the reproducibility of the result and to obtain the statistical histograms of the photovoltaic parameters. The EQE measurements were carried out on an ORIEL Intelligent Quantum Efficiency (IQE) 200TM Measurement system established with a tunable light source. To evaluate the UV‐light stability of the devices, a high‐voltage mercury lamp irradiation (500 W, China Education Au‐light) with a 365 nm cutoff filter (QD 365 nm, China Education Au‐light) was used.

SEM and EDS measurements were attained via a field‐emission scanning electron microscope (FEI Apero). AFM images were carried out on a XE‐7 scanning probe microscope in noncontact mode (Park systems, Korea). XPS measurements were performed on a Thermo ESCALAB 250 instrument with a monochromatized Al K*α* X‐ray source in vacuum. The XRD patterns were obtained on a Rigaku SmartLab X‐ray diffractometer with Cu‐K*α* radiation (0.154 nm). UV–vis spectroscopy was recorded on a UV–vis–NIR 3600 spectrometer (Shimadzu, Japan). The steady‐state PL spectra were recorded by employing an Edinburgh Instruments FLS920 fluorescence spectrometer with an excitation wavelength of 460 nm. The TRPL spectra were measured via the time‐correlated single‐photon counting method with a Picoquant Gmbh Solea Supercontinuum Laser. A picosecond pulsed diode laser at 543 nm with a pulse width of 10^4^ ps was used as the excitation source. Impedance spectroscopic measurements (EIS) were performed in dark using an electrochemical workstation (Autolab 320, Metrohm, Switzerland) with a frequency range from 1 Hz to 1 MHz under 1.0 V. AC 20 mV perturbation was applied with a frequency from 1 MHz to 1 Hz. The obtained impedance spectra were fitted with Z‐View software (v2.8b, Scribner Associates, USA). All the measurements were carried out in ambient surroundings. The GIXRD measurements were performed at the BL14B1 beamline of Shanghai Synchrotron Radiation Facility (SSRF) using X‐ray with a wavelength of 1.24 Å. The solid‐state ^19^F NMR measurements were carried out on Bruker Advance III 400WB equipped with an Ascend 600 MHz magnet. The 2D‐GIXRD patterns were obtained by a MarCCD detector mounted vertically at a distance of around 274 mm from the sample with an exposure time of 50 s at a grazing incidence angle of 0.2°. The 2D‐GIXRD patterns were analyzed using software Fit 2D and displayed in scattering vector *q* coordinates with *q* = 4*π*sin *θ*/*λ*, where *θ* is half of the diffraction angle, and *λ* is the wavelength of incident X‐ray. The DLTS measurements were carried out with a Phystech FT‐1230 HERA DLTS system at various temperatures with reverse bias. Temperature scans were made between 120 and 430 K, at a heating rate of 2 K min^−1^. The capacitances of samples were measured by using a Boonton 7200 Phystech capacitance meter with a 1 MHz capacitance meter. The reverse bias, pulse height, pulse width, and period width set as −0.6 V, 0.6 V, 10 ms, and 20 ms, respectively. The measurements were conducted two times to ensure reproducibility. The pulse frequency for the voltage was 100 MHz.

## Conflict of Interest

The authors declare no conflict of interest.

## Supporting information

Supporting Information

## Data Availability

Research data are not shared.

## References

[1] Y. Hou , E. Aydin , M. De Bastiani , C. X. Xiao , F. H. Isikgor , D. J. Xue , B. Chen , H. Chen , B. Bahrami , A. H. Chowdhury , A. Johnston , S. W. Baek , Z. R. Huang , M. Y. Wei , Y. T. Dong , J. Troughton , R. Jalmood , A. J. Mirabelli , T. G. Allen , E. Van Kerschaver , M. I. Saidaminov , D. Baran , Q. Q. Qiao , K. Zhu , S. De Wolf , E. H. Sargent , Science 2020, 367, 1135. 32139544 10.1126/science.aaz3691

[2] NREL , Efficiency chart, https://www.nrel.gov/pv/cell‐efficiency.html (accessed: June 2020).

[3] M. Vasilopoulou , A. Fakharuddin , A. G. Coutsolelos , P. Falaras , P. Argitis , A. Yusoff , M. K. Nazeeruddin , Chem. Soc. Rev. 2020, 49, 4496. 32495754 10.1039/c9cs00733d

[4] Y. Wang , T. Wu , J. Barbaud , W. Kong , D. Cui , H. Chen , X. Yang , L. Han , Science 2019, 365, 687. 31416961 10.1126/science.aax8018

[5] F. Guo , S. Qiu , J. Hu , H. Wang , B. Cai , J. Li , X. Yuan , X. Liu , K. Forberich , C. J. Brabec , Y. Mai , Adv. Sci. 2019, 6, 1901067. 10.1002/advs.201901067 PMC6724353 31508290

[6] K. Huang , Y. Peng , Y. Gao , J. Shi , H. Li , X. Mo , H. Huang , Y. Gao , L. Ding , J. Yang , Adv. Energy Mater. 2019, 9, 1901419.

[7] W. Hu , S. Yang , S. Yang , Trends Chem. 2020, 2, 148.

[8] F. Wang , Y. Cao , C. Chen , Q. Chen , X. Wu , X. Li , T. Qin , W. Huang , Adv. Funct. Mater. 2018, 28, 1803753.

[9] H. Tan , A. Jain , O. Voznyy , X. Lan , F. P. Garcia de Arquer , J. Z. Fan , R. Quintero‐Bermudez , M. Yuan , B. Zhang , Y. Zhao , F. Fan , P. Li , L. N. Quan , Y. Zhao , Z. H. Lu , Z. Yang , S. Hoogland , E. H. Sargent , Science 2017, 355, 722. 28154242 10.1126/science.aai9081

[10] H. Zhou , Q. Chen , G. Li , S. Luo , T. B. Song , H. S. Duan , Z. Hong , J. You , Y. Liu , Y. Yang , Science 2014, 345, 542. 25082698 10.1126/science.1254050

[11] W. Hu , W. Zhou , X. Lei , P. Zhou , M. Zhang , T. Chen , H. Zeng , J. Zhu , S. Dai , S. Yang , S. Yang , Adv. Mater. 2019, 31, 1806095. 10.1002/adma.201806095 30633399

[12] B. Wang , M. Zhang , X. Cui , Z. Wang , M. Rager , Y. Yang , Z. Zou , Z. L. Wang , Z. Lin , Angew. Chem., Int. Ed. 2020, 132, 1628. 10.1002/anie.201910471 31664750

[13] D. Yang , R. Yang , K. Wang , C. Wu , X. Zhu , J. Feng , X. Ren , G. Fang , S. Priya , S. F. Liu , Nat. Commun. 2018, 9, 3239. 30104663 10.1038/s41467-018-05760-x PMC6089874

[14] C. Zhen , T. Wu , R. Chen , L. Wang , G. Liu , H. M. Cheng , ACS Sustainable Chem. Eng. 2019, 7, 4586.

[15] P. Chen , Z. Wang , S. Wang , M. Lyu , M. Hao , M. Ghasemi , M. Xiao , J. H. Yun , Y. Bai , L. Wang , Nano Energy 2020, 69, 104392.

[16] N. Li , S. Tao , Y. Chen , X. Niu , C. K. Onwudinanti , C. Hu , Z. Qiu , Z. Xu , G. Zheng , L. Wang , Y. Zhang , L. Li , H. Liu , Y. Lun , J. Hong , X. Wang , Y. Liu , H. Xie , Y. Gao , Y. Bai , S. Yang , G. Brocks , Q. Chen , H. Zhou , Nat. Energy 2019, 4, 408.

[17] J. Pan , G. Liu , G. Q. Lu , H. M. Cheng , Angew. Chem., Int. Ed. 2011, 50, 2133. 10.1002/anie.201006057 21344568

[18] T. R. Gordon , M. Cargnello , T. Paik , F. Mangolini , R. T. Weber , P. Fornasiero , C. B. Murray , J. Am. Chem. Soc. 2012, 134, 6751. 22444667 10.1021/ja300823a

[19] S. Liu , J. Yu , M. Jaroniec , J. Am. Chem. Soc. 2010, 132, 11914. 20687566 10.1021/ja105283s

[20] H. Park , W. Choi , J. Phys. Chem. B 2004, 108, 4086. 10.1021/jp049789g 18950128

[21] S. Wang , X. Liu , L. Wang , Q. Wen , N. Du , J. Huang , RSC Adv. 2017, 7, 16078.

[22] D. N. Pei , L. Gong , A. Y. Zhang , X. Zhang , J. J. Chen , Y. Mu , H. Q. Yu , Nat. Commun. 2015, 6, 8696. 26493365 10.1038/ncomms9696 PMC4846326

[23] S. Liu , J. Yu , M. Jaroniec , Chem. Mater. 2011, 23, 4085.

[24] A. Czoska , S. Livraghi , M. Chiesa , E. Giamello , S. Agnoli , G. Granozzi , E. Finazzi , C. D. Valentin , G. Pacchioni , J. Phys. Chem. C 2008, 112, 8951.

[25] X. Yao , J. Liang , Y. Li , J. Luo , B. Shi , C. Wei , D. Zhang , B. Li , Y. Ding , Y. Zhao , X. Zhang , Adv. Sci. 2017, 4, 1700008. 10.1002/advs.201700008 PMC5644234 29051848

[26] K. M. Lee , M. Y. Hou , V. Suryanarayanan , M. C. Wu , ChemSusChem 2018, 11, 3234. 30022626 10.1002/cssc.201801249

[27] A. Huang , L. Lei , J. Zhu , Y. Yu , Y. Liu , S. Yang , S. Bao , X. Cao , P. Jin , ACS Appl. Mater Interfaces 2017, 9, 2016. 28072509 10.1021/acsami.6b14040

[28] Y. Lv , R. Yuan , B. Cai , B. Bahrami , A. H. Chowdhury , C. Yang , Y. Wu , Q. Qiao , S. Liu , W. Zhang , Angew. Chem., Int. Ed. 2020, 59, 11969. 10.1002/anie.201915928 32293091

[29] S. Zhu , S. Liang , Q. Gu , L. Xie , J. Wang , Z. Ding , P. Liu , Appl. Catal., B 2012, 119–120, 146.

[30] P. You , Z. Liu , Q. Tai , S. Liu , F. Yan , Adv. Mater. 2015, 27, 3632. 25969400 10.1002/adma.201501145

[31] G. Zheng , C. Zhu , J. Ma , X. Zhang , G. Tang , R. Li , Y. Chen , L. Li , J. Hu , J. Hong , Q. Chen , X. Gao , H. Zhou , Nat. Commun. 2018, 9, 2793. 30022027 10.1038/s41467-018-05076-w PMC6052040

[32] Y. Zhang , P. Wang , M. C. Tang , D. Barrit , W. Ke , J. Liu , T. Luo , Y. Liu , T. Niu , D. M. Smilgies , Z. Yang , Z. Liu , S. Jin , M. G. Kanatzidis , A. Amassian , S. F. Liu , K. Zhao , J. Am. Chem. Soc. 2019, 141, 2684. 30648861 10.1021/jacs.8b13104

[33] S. Yuan , F. Qian , S. Yang , Y. Cai , Q. Wang , J. Sun , Z. Liu , S. Liu , Adv. Funct. Mater. 2019, 29, 1807850.

[34] M. Shibakami , A. Sekiya , Bull. Chem. Soc. Jpn. 1993, 66, 315.

[35] M. M. Zhang , W. P. Hu , Y. B. Shang , W. R. Zhou , W. F. Zhang , S. F. Yang , Sol. RRL 2020, 4, 2000113.

[36] M. J. Paik , Y. Lee , H.‐S. Yun , S.‐U. Lee , S.‐T. Hong , S. I. Seok , Adv. Energy Mater. 2020, 10, 2001799.

[37] S. Song , G. Kang , L. Pyeon , C. Lim , G.‐Y. Lee , T. Park , J. Choi , ACS Energy Lett. 2017, 2, 2667.

[38] Z. Ni , C. Bao , Y. Liu , Q. Jiang , W. Q. Wu , S. Chen , X. Dai , B. Chen , B. Hartweg , Z. Yu , Z. Holman , J. Huang , Science 2020, 367, 1352. 32193323 10.1126/science.aba0893

[39] M. Zhang , M. Ye , W. Wang , C. Ma , S. Wang , Q. Liu , T. Lian , J. Huang , Z. Lin , Adv. Mater. 2020, 32, 2000999. 10.1002/adma.202000999 32406152

[40] W. Hu , X. He , Z. Fang , W. Lian , Y. Shang , X. Li , W. Zhou , M. Zhang , T. Chen , Y. Lu , L. Zhang , L. Ding , S. Yang , Nano Energy 2020, 68, 104362.

[41] Y. C. Choi , D. U. Lee , J. H. Noh , E. K. Kim , S. I. Seok , Adv. Funct. Mater. 2014, 24, 3587.

[42] N. Liu , C. Yam , Phys. Chem. Chem. Phys. 2018, 20, 6800. 29473061 10.1039/c8cp00280k

[43] D. Luo , R. Su , W. Zhang , Q. Gong , R. Zhu , Nat. Rev. Mater. 2019, 5, 44.

[44] J. Chen , X. Zhao , S. G. Kim , N. G. Park , Adv. Mater. 2019, 31, 1902902. 10.1002/adma.201902902 31402565

[45] Y. C. Shih , Y. B. Lan , C. S. Li , H. C. Hsieh , L. Wang , C. I. Wu , K. F. Lin , Small 2017, 13, 1604305. 10.1002/smll.201604305 28401749

[46] W. Hui , Y. Yang , Q. Xu , H. Gu , S. Feng , Z. Su , M. Zhang , J. Wang , X. Li , J. Fang , F. Xia , Y. Xia , Y. Chen , X. Gao , W. Huang , Adv. Mater. 2019, 32, 1906374. 10.1002/adma.201906374 31799762

[47] W. Chen , S. Zhang , Z. Liu , S. Wu , R. Chen , M. Pan , Z. Yang , H. Zhu , S. Liu , J. Tang , J. Li , W. Chen , Sol. RRL 2019, 3, 1900346.

[48] W. Zhou , D. Li , Z. Xiao , Z. Wen , M. Zhang , W. Hu , X. Wu , M. Wang , W. H. Zhang , Y. Lu , S. Yang , S. Yang , Adv. Funct. Mater. 2019, 29, 1901026.

[49] R. Wang , M. Mujahid , Y. Duan , Z. K. Wang , J. J. Xue , Y. Yang , Adv. Funct. Mater. 2019, 29, 25.

[50] V. Zardetto , F. di Giacomo , H. Lifka , M. A. Verheijen , C. H. Weijtens , L. E. Black , S. Veenstra , W. M. Kessels , R. Andriessen , M. Creatore , Adv. Mater. Interfaces 2018, 5, 1701456.

