# Supplementary material for: In Situ Surface Fluorination of TiO2 Nanocrystals Reinforces Interface Binding of Perovskite Layer for Highly Efficient Solar Cells with Dramatically Enhanced Ultraviolet‐Light Stability
Source: Adv Sci (Weinh). 2021 Mar 13;8(10):2004662. doi: 10.1002/advs.202004662 (PMC8132056; doi:10.1002/advs.202004662)
Supplement: Supplementary file 1 — Supporting Information [file ADVS-8-2004662-s001.pdf]

WILEY-VCH

© 2021 Wiley-VCH GmbH

ADVANCED SCIENCE
Open Access

Supporting Information

for *Adv. Sci.*, DOI: 10.1002/advs.202004662

In-situ Surface Fluorination of TiO2 Nanocrystals Reinforces Interface Binding of Perovskite Layer for Highly Efficient Solar Cells with Dramatically Enhanced Ultraviolet-Light Stability

*Wanpei Hu, Zhiling Wen, Xin Yu, Peisen Qian, Weitao Lian, Xingcheng Li, Yanbo Shang, Xiaojun Wu, Tao Chen, Yalin Lu, Mingtai Wang, and Shangfeng Yang**

© 2021 Wiley-VCH GmbH

Supporting Information

In-situ Surface Fluorination of TiO_2 Nanocrystals Reinforces Interface Binding of Perovskite Layer for Highly Efficient Solar Cells with Dramatically Enhanced Ultraviolet-Light Stability

*Wanpei Hu, Zhiling Wen, Xin Yu, Peisen Qian, Weitao Lian, Xingcheng Li, Yanbo Shang, Xiaojun Wu, Tao Chen, Yalin Lu, Mingtai Wang, and Shangfeng Yang**

Contents

S1. Transmission electron microscopic (TEM) images of different TiO_2 nanocrystals..... 3

S2. The full width at half-maximum (FWHM) of the characteristic diffraction peaks of different TiO_2. 3

S3. XPS result of different TiO_2 nanocrystals. 4

S4. Atomic structures and projected density of states of the perfect and defective anatase TiO_2 (001) surfaces with or without F. 5

S5. Optical transmittance spectra of different TiO_2 films deposited on ITO substrates. . 7

S6. Diffuse reflectance spectra of different TiO_2 nanocrystals. 8

S7. SCLC carrier mobilities of different TiO_2 films 8

S8. Photocatalytic activity of different TiO_2 sintered at 600 ℃ 9

S9. SEM and AFM morphologies of different TiO_2 flims 9

S10. Histograms of grain size distributions of perovskite films deposited on different TiO_2 substrates 10

S11. AFM images of perovskite films deposited on different TiO_2 substrates 10

S12. The integrated intensities of perovskite films deposited on different TiO_2 films obtained by 2D-GIXRD profiles. 10

S13. Evidence for the interactions between the bonded F^- anion with perovskite. 11

S14. Optimization of the F:Ti molar ratios according to the ETL performance. 11

S15. Stabilized photocurrent density and power output of the device based on the pristine TiO_2 ETL. 13

S16. Photovoltaic parameters under forward and reverse scans. 13

S17. Comparison of PCE and V_{oc} values of the rigid PHJ-PSC devices based on low-temperature solution-processed TiO_2 ETLs reported in literatures. 14

S18. Box charts of the statistical photovoltaic parameters of PSCs based on different TiO_2 ETLs. 16

S19. Photovoltaic parameters of the flexible PHJ-PSC devices based on different TiO_2 ETLs. 16

S20. Comparison of PCE and V_{oc} values of the flexible PHJ-PSCs based on LT-TiO_2 ETLs reported in literatures 17

S21. Work functions of different TiO_2 ETLs and CsFAMA perovskite film. 19

S22. Electron trap-state densities of perovskite films deposited on different TiO_2 ETLs. 19

S23. Deep level defects of devices based on different TiO_2 ETLs estimated by DLTS. 20

S24. Atomic structures and projected density of states of the main antisite defects of perovskite before and after F incorporation. 22

S25. Analysis of time-resolved photoluminescence (TRPL) spectra of the perovskite films deposited on different TiO_2 ETLs. 25

S26. Fitting parameters for EIS data. 26

S27. Ambient stabilities of the devices based on different TiO_2 ETLs. 27

Reference 27

S1. Transmission electron microscopic (TEM) images of different TiO_2 nanocrystals.

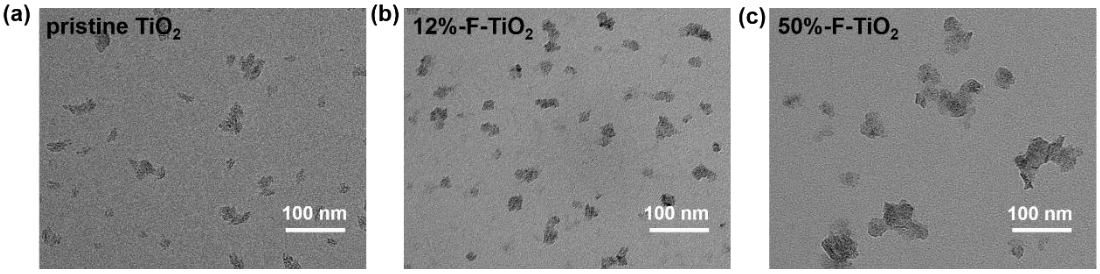

Figure S1. Transmission electron microscopic (TEM) images of the pristine TiO_2 (a), 12%-F-TiO_2 (b), and 50%-F-TiO_2 nanocrystals (c) dispersed in ethanol.

S2. The full width at half-maximum (FWHM) of the characteristic diffraction peaks of different TiO_2.

Table S1. The full width at half-maximum (FWHM) and the corresponding crystal sizes[a] of the (101), (200) and (004) diffraction peaks of different TiO_2 along with the crystal shapes.

Sample	(101)		(004)		(200)		Crystal shape
	FWHM	$D_{[101]}$ (nm)	FWHM	$D_{[004]}$ (nm)	FWHM	$D_{[200]}$ (nm)	
pristine TiO_2	0.839	9.69	0.772	10.86	0.845	10.27	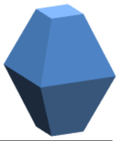
4%-F-TiO_2	0.778	10.45	0.828	10.13	0.812	10.69	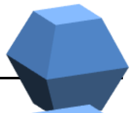
12%-F-TiO_2	0.725	11.22	0.857	9.79	0.779	11.14	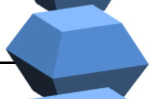
50%-F-TiO_2	0.709	11.47	1.153	7.27	0.586	14.81	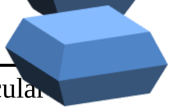

[a] Scherrer's equation: $D = K\lambda/\beta \cos \theta$, where D is average thickness of grain perpendicular [illegible] plane (nm), K is geometry factor, which is 0.9 in this case, λ is the wavelength of X-ray, β is the half-peak width, and θ is the diffraction angle.

S3. XPS result of different TiO_2 nanocrystals.

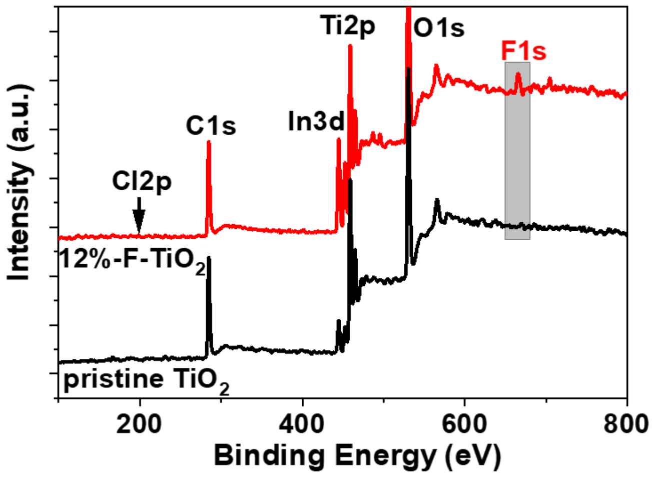

Figure S2. XPS survey spectra the pristine TiO_2 and 12%-F-TiO_2 films.

Table S2. The atomic ratio of F: Ti of the pristine TiO_2 and different F-TiO_2 films calculated from the XPS results.

Sample	Atomic ratio (%)				
	C	Ti	O	F	F:Ti
pristine TiO_2	45.0	16.6	38.4	0	0
4%-F-TiO_2	45.8	15.7	38.1	0.35	0.022
12%-F-TiO_2	39.4	16.2	43.6	0.81	0.050
50%-F-TiO_2	41.8	15.9	41.3	1.05	0.066

Table S3. Positions, areas, and area ratios of the deconvoluted O1s peaks for the pristine and 12%-F-TiO_2 powders.

		**pristine TiO_2**	**12%-F-TiO_2**
O_M	**position**	530.15eV	530.20eV
	**area**	48889.8	53964.3
O_V	**position**	531.12eV	531.11eV
	**area**	14057.4	9911.7
O_{OH}	**position**	531.89eV	531.89eV
	**area**	26970.5	16431.1
O_M /(O_M + O_V + O_{OH}) area ratio (%)		54.4	67.2
O_V /(O_M + O_V + O_{OH}) area ratio (%)		15.6	12.3
O_{OH} /(O_M + O_V + O_{OH}) area ratio (%)		30.0	20.5

S4. Atomic structures and projected density of states of the perfect and defective anatase TiO_2 (001) surfaces with or without F.

Computational details: All calculations were carried out using the Vienna ab Initio Simulation Package (VASP)[S1] based on the density function theory (DFT) with projector augmented wave potentials.[S2] Exchange and correlation were treated in the generalized gradient approximation (GGA) of Perdew Burke Ernzerhof (PBE).[3] Taking into account of the long-range effect of van der Waals (vdW) force, the vdW interactions were added with the DFT-D3 (BJ) method of Grimme.[S4] The simulations were carried out with a 500 eV kinetic energy cutoff for a plane wave basis set, and the atomic structures were fully optimized until the forces were smaller than |0.05|eV/Å and energy change was smaller than 10^{-5} eV.

While studying the effect of fluoride ions on the surface of TiO_2 (001) surface, the lattice constants of the fully optimized bulk anatase TiO_2 structure are a=b=3.8048Å, c=9.7097Å which is close to Experimental results[S5]. And the

O-terminated surface model is built by a (2×2) supercell of aTiO_2(001) surface with a 15Å vacuum layer, it contains four O-Ti bilayers and the bottom two layers were fixed during calculations. 4x4x1 Monkhorst-Pack k-point mesh was adopted for Brillouin-zone sampling for all calculations related to TiO_2. To accurately describe the electronic structure of aTiO_2 surface, the Hubbard U correction[S6] for the 3d electrons of Ti atoms was used and the U(Ti-3d)=7eV under which the band gap of the bulk aTiO_2 (3.233eV) is similar to experimental value[S7].

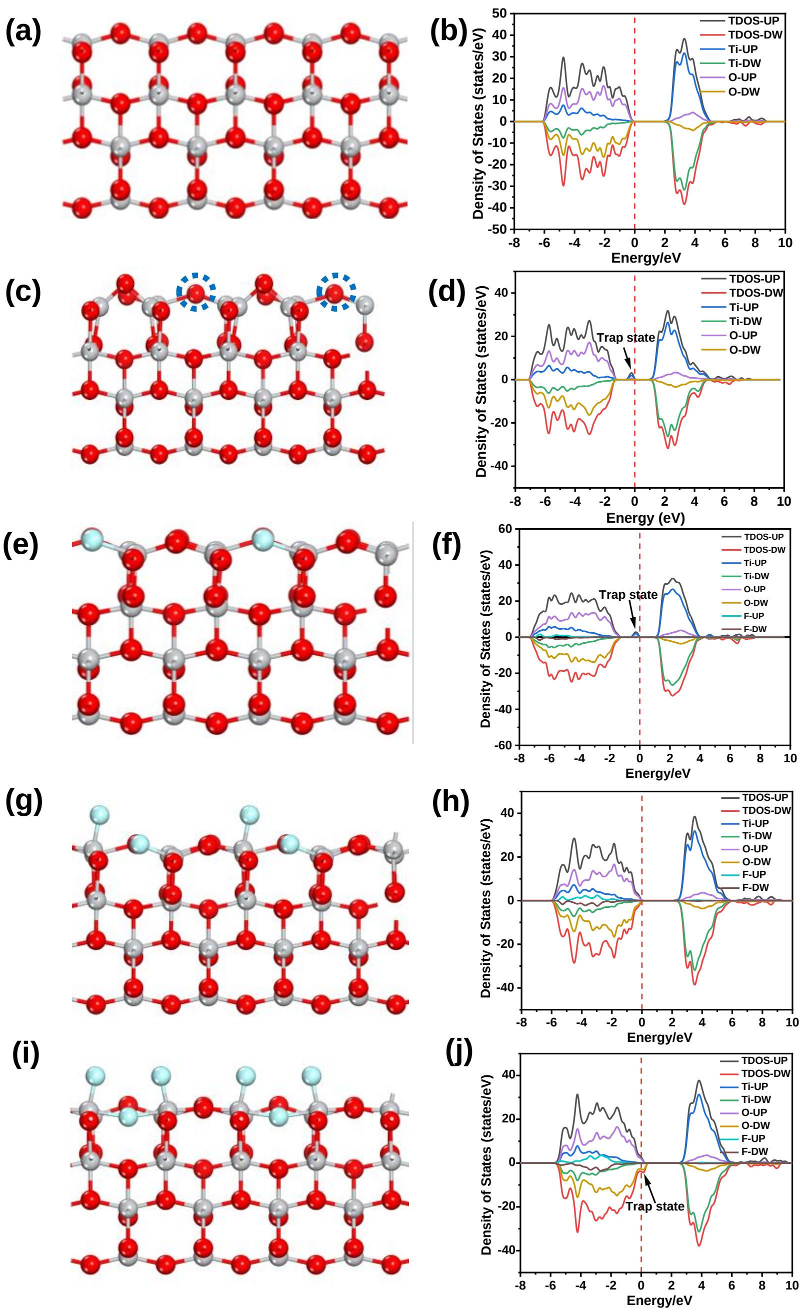

Figure S3. Atomic structures (top) and projected density of states (PDOS) (bottom) of the perfect anatase TiO_2 (001) surface (a, b), the anatase TiO_2 (001) surface with oxygen vacancy (O_v) (c, d); With one F atom adjacent to the oxygen vacancy (1F-O_v) (e, f); With two F atoms adjacent to the oxygen vacancy (2F-O_v) (g, h); With three F atoms adjacent to the oxygen vacancy (3F-O_v) (i, j). The blue dotted circle represents the O_v. The red, gray, and cyan spheres represent O, Ti and F atoms respectively.

S5. Optical transmittance spectra of different TiO_2 films deposited on ITO substrates.

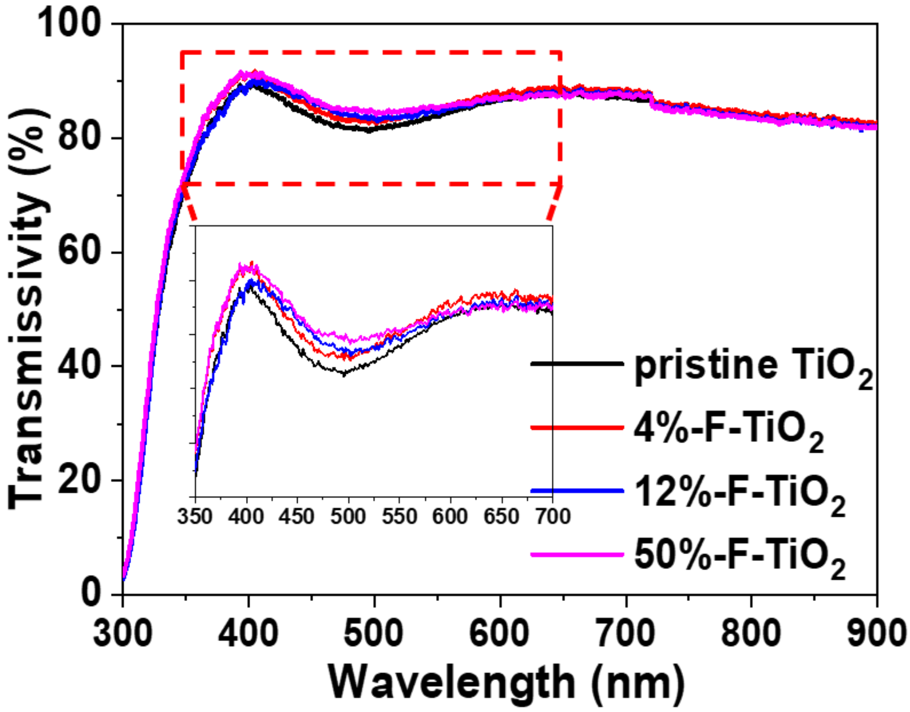

Figure S4. Optical transmittance spectra of different TiO_2 films deposited on ITO substrates.

S6. Diffuse reflectance spectra of different TiO_2 nanocrystals.

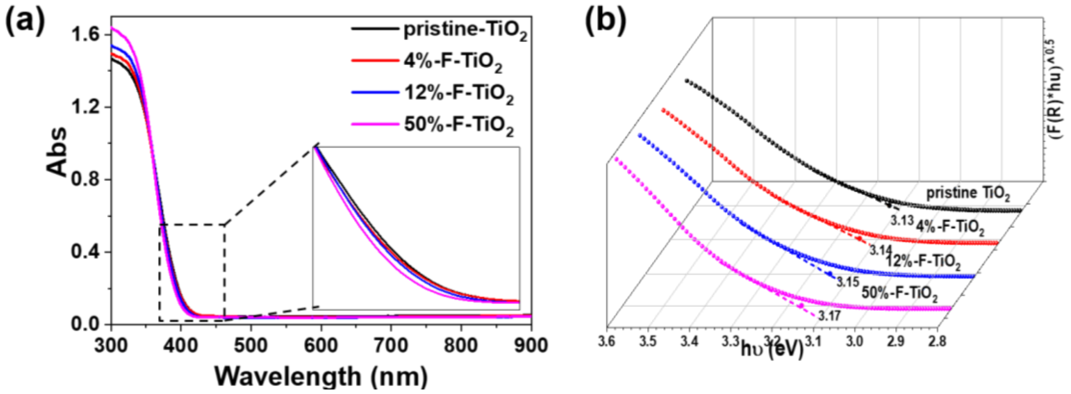

Figure S5. (a) Diffuse reflectance spectra of different TiO_2 nanocrystal powders; (b) The optical bandgaps of different TiO_2 nanocrystals estimated by using the Kubelka-Munk formula (based on the point of intersection of the tangent line and the horizontal axis).

S7. SCLC carrier mobilities of different TiO_2 films.

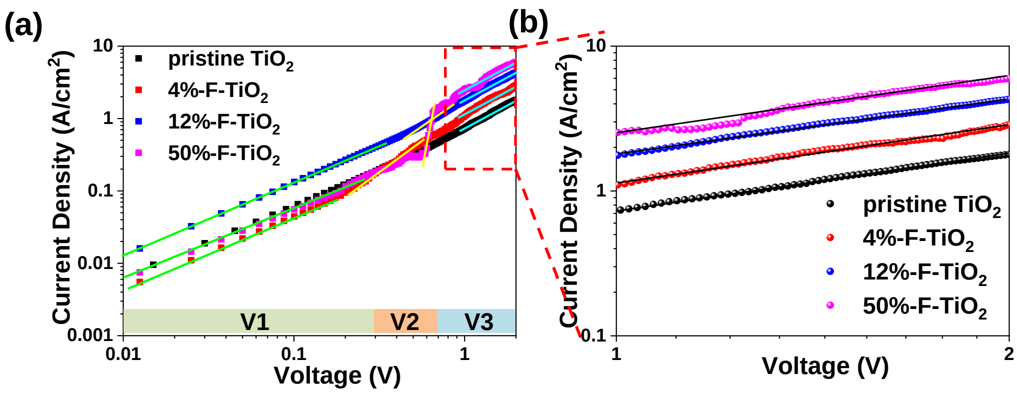

Figure S6. Dark current-voltage curves of the electron-only devices utilized for estimating the TiO_2 SCLC electron mobility: (a) the complete J-V curve; (b) the J-V curve from SCLC region. V1: the Ohmic region, V2: the trap-filled limited region, V3: the SCLC region.

Table S4. SCLC carrier mobilities of different TiO_2 films.

Films	Thickness (nm)	Electron mobility (μ_e) ($cm^2V^{-1}S^{-1}$)
pristine TiO_2	80	0.77×10^{-4}
4%-F-TiO_2	80	1.21×10^{-4}
12%-F-TiO_2	80	1.92×10^{-4}
50%-F-TiO_2	100	5.29×10^{-4}

S8. Photocatalytic activity of different TiO_2 sintered at 600 ℃

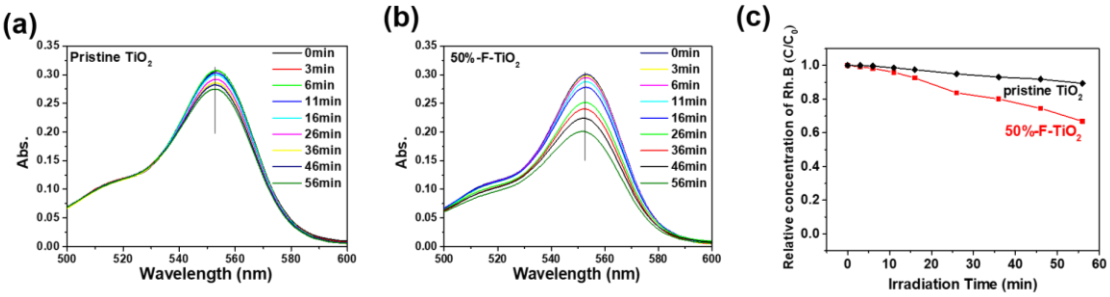

Figure S7. UV-vis spectra of the RhB dye in different aqueous TiO_2 dispersions as a function of the irradiation time based on the pristine TiO_2 (a) and 50%-F-TiO_2 sintered at 600 ^{o}C in air for 3h (b). (c) Photocatalytic degradation of RhB over the different TiO_2 powders after calcination at 600 ℃ in air for 3h under the simulated sunlight light. C and C_0 denote the reaction and absorption equilibrium concentrations of RhB in the system.

S9. SEM and AFM morphologies of different TiO_2 flims

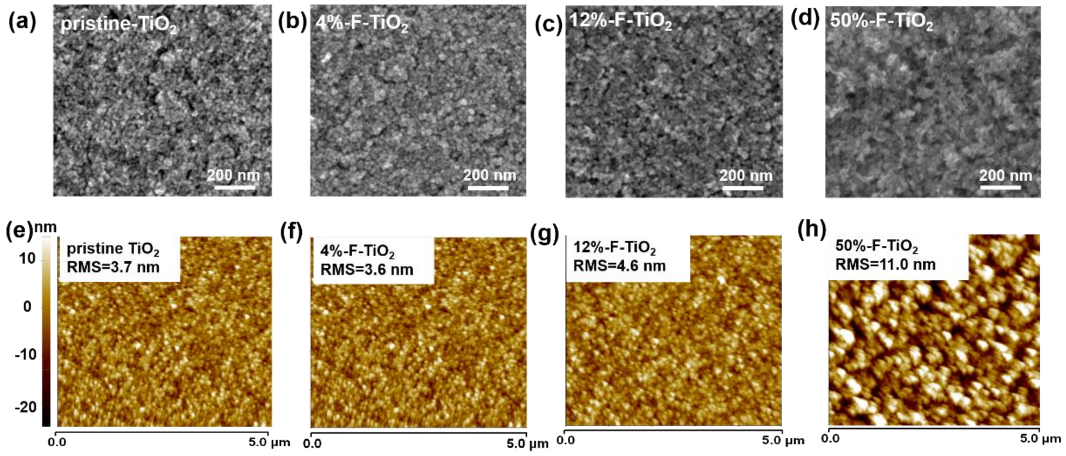

Figure S8. Surface topographic SEM images (top) and AFM height images (bottom) of different TiO_2 flims. (a, e) the pristine TiO_2, 4-F-TiO_2 (b, f), 12%-F-TiO_2 (c, g) and 50%-F-TiO_2 (d, h).

S10. Histograms of grain size distributions of perovskite films deposited on different TiO_2 substrates.

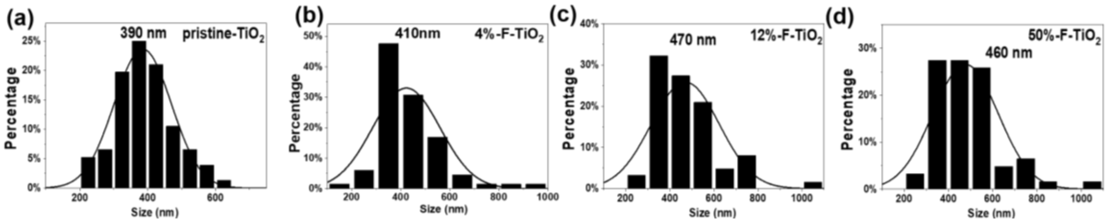

Figure S9. Histograms of grain size distributions of perovskite films spin-coated on the pristine TiO_2 (a), 4%-F-TiO_2 (b), 12%-F-TiO_2 (c) and 50%-F-TiO_2 (d) estimated from the SEM images using Nano measurer 1.2 software.

S11. AFM images of perovskite films deposited on different TiO_2 substrates.

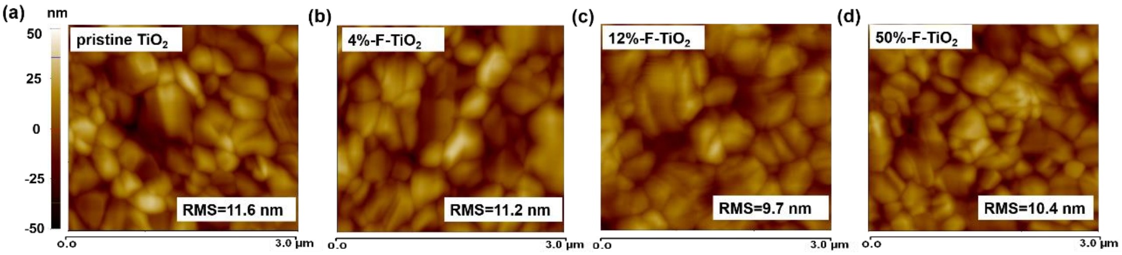

Figure S10. AFM height images of perovskite films deposited on different TiO_2 substrates: pristine TiO_2 (a), 4%-F-TiO_2 (b) , 12%-F-TiO_2 (c) and 50%-F-TiO_2 (d).

S12. The integrated intensities of perovskite films deposited on different TiO_2 films obtained by 2D-GIXRD profiles.

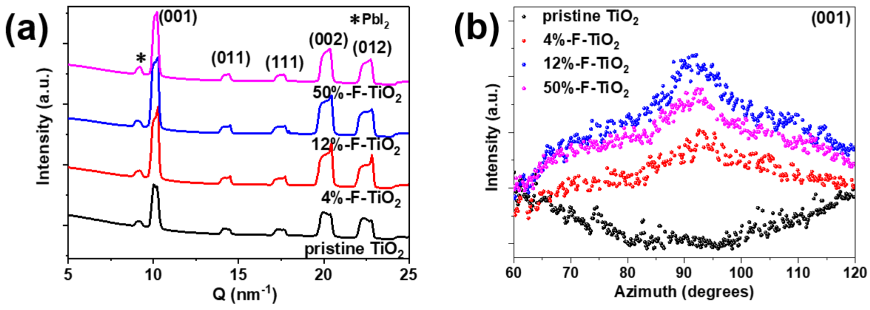

Figure S11. The radially integrated intensity profiles (a) and the azimuthally integrated intensity plots along the ring at q ~10 nm^{-1} (b) for the perovskite films deposited on different TiO_2 substrates obtained from 2D-GIXRD profiles.

S13. Evidence for the interactions between the bonded F^- anion with perovskite.

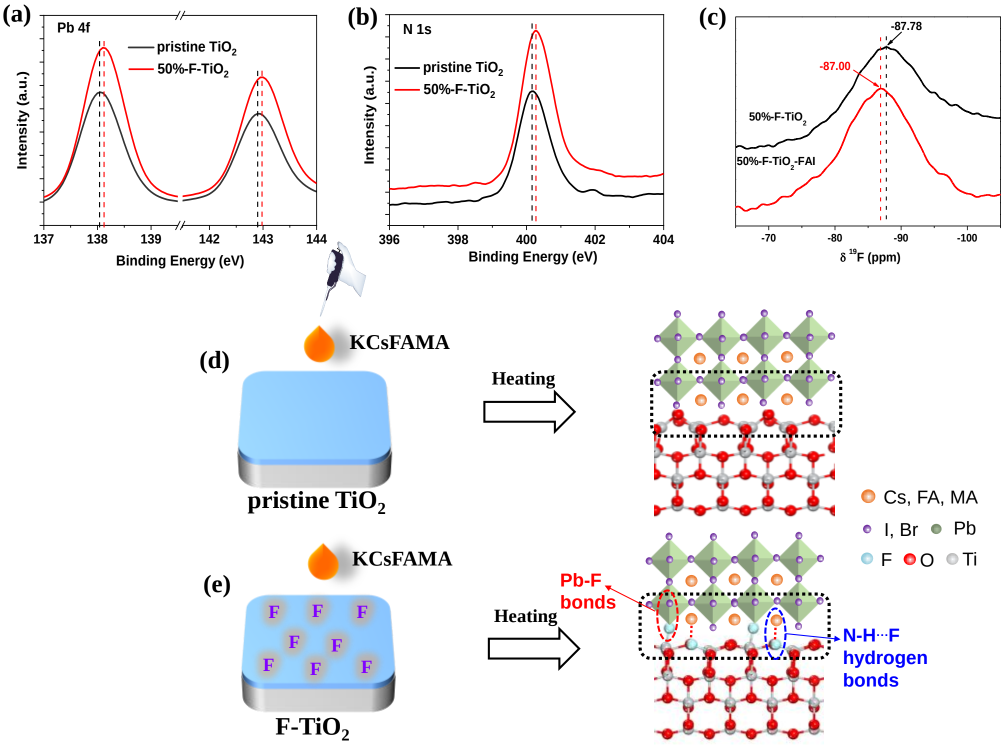

Figure S12. Pb 4f (a) and N 1s (b) XPS spectra of perovskite films covered by TiO_2 layer spin-coated from the isopropanol suspension of 50%-F-TiO_2 and pristine TiO_2 nanocrystals. (c) Solid state ^{19}F NMR spectra of 50%-F-TiO_2 powder and 50%-F-TiO_2/FAI mixture powder. (d-e) Schematic illustration of the fabrication process and proposed formation mechanisms of perovskite films based on different TiO_2 substrates.

S14. Optimization of the F:Ti molar ratios according to the ETL performance.

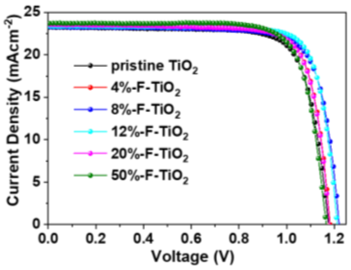

Figure S13. J-V curves of the champion devices based on different TiO_2 ETLs. The scanning direction is from open-circuit voltage to short circuit (reverse) with a scan rate of 0.1 V/s.

Table S5. Photovoltaic parameters of PSC devices based on different TiO_2 ETLs under one sun illumination (AM 1.5G, 100 mA cm^{-2}).

Molar ratio of F:Ti [a]	Devices		V_{oc} (V)	J_{sc} (mA/cm^2)	FF (%)	PCE (%)	R_s [c] (Ω•cm^2)	R_{sh} [c] (Ω•cm^2)
0%	pristine TiO_2	Average [b]	1.16 ±0.02	22.35 ±0.79	73.86 ±2.82	19.17 ±1.11	4.58	2272.36
		Champion	1.17	23.23	77.33	21.09	3.79	3578.55
4%	4%-F-TiO_2	Average [b]	1.16 ±0.02	22.74 ±0.74	75.11 ±2.20	19.87 ±1.05	4.28	2919.47
		Champion	1.18	23.47	78.69	21.80	3.25	3433.31
8%	8%-F-TiO_2	Average [b]	1.17 ±0.02	22.78 ±0.76	75.64 ±2.49	20.17 ±1.11	4.06	3731.24
		Champion	1.22	23.28	77.93	22.12	3.4	4255.34
12%	12%-F-TiO_2	Average [b]	1.17 ±0.02	22.90±0.61	77.03 ±1.90	20.66 ±0.97	3.89	3706.49
		Champion	1.21	23.36	80.25	22.68	3.32	8534.90
20%	20%-F-TiO_2	Average [b]	1.17 ±0.02	22.92 ±0.80	74.73 ±2.51	20.01 ±1.08	4.15	3055.05
		Champion	1.19	23.37	78.72	21.87	3.31	4602.49
50%	50%-F-TiO_2	Average [b]	1.15±0.03	22.96±0.68	74.89±2.38	19.79±0.97	4.08	2740.63
		Champion	1.16	23.71	78.05	21.55	3.95	9206.54

[a] the molar ratio of F^- relative to Ti^{4+} in the raw solution; [b] Averaged over 60 devices fabricated independently; [c] R_s and R_{sh} are given by the PCE measurement system.

S15. Stabilized photocurrent density and power output of the device based on the pristine TiO_2 ETL.

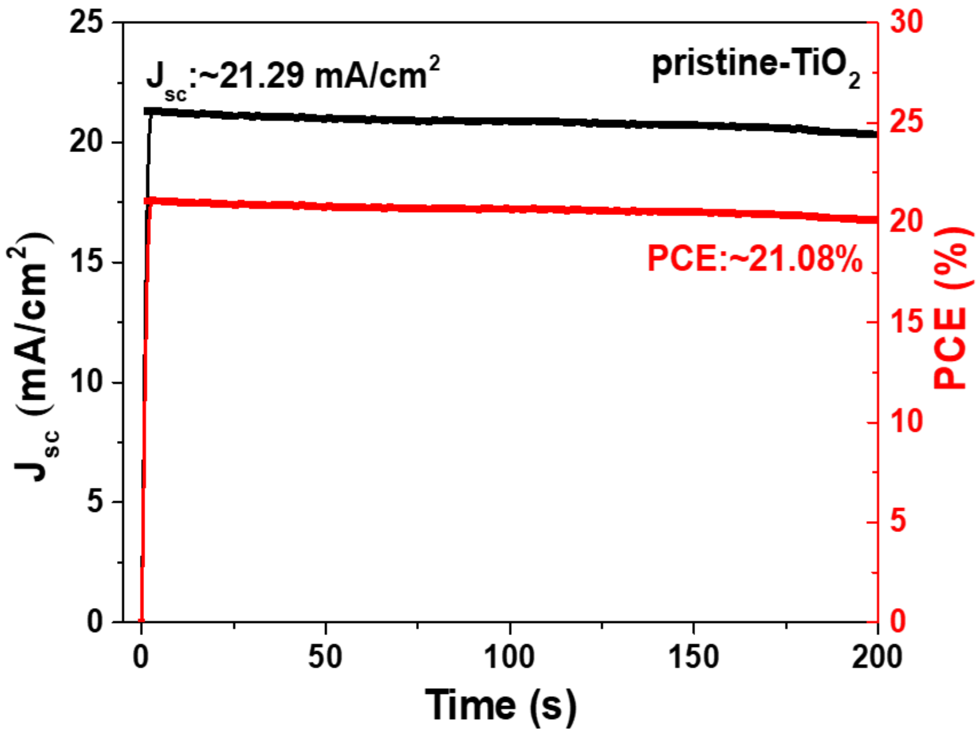

Figure S14. The stabilized photocurrent and power output of the PSCs based on pristine TiO_2 measured at the maximum power point (0.99 V).

S16. Photovoltaic parameters under forward and reverse scans.

Table S6. Photovoltaic parameters of the devices in different scan directions with 0.1 V/s scan rate.

ETL	Scan direction	V_{oc} (V)	J_{sc} (mA/cm^2)	FF (%)	PCE (%)	Hystereis index [a] (%)
pristine TiO_2	reverse	1.17	21.75	78.24	19.91	1.9
	forward	1.18	21.74	79.09	20.29	
12%-F-TiO_2	reverse	1.19	22.99	79.92	21.79	1.7
	forward	1.18	23.52	77.50	21.42	

[a] Hystersis index = [PCE(reverse) - PCE(forward)]/PCE(reverse)

S17. Comparison of PCE and V_{oc} values of the rigid PHJ-PSC devices based on low-temperature solution-processed TiO_2 ETLs reported in literatures.

Table S7. A list of photovoltaic parameters of the PHJ-PSC devices based on low-temperature solution-processed TiO_2 ETLs reported in literatures.

Structure	Voc (V)	Jsc ($mAcm^{-2}$)	FF (%)	PCE (%)	Temp reture (℃)	Method of TiO_2 preparation	Remark	Ref.
ITO/TiO_2-Cl/$Cs_{0.05}FA_{0.81}MA_{0.14}PbI_{2.55}Br_{0.45}$/Spiro-OMeTAD/Au	1.19	22.30	80.6	21.40	150	Nonhydrolytic sol-gel reaction	Contact passivation	[S8]
ITO/TiO_2-Cl/$(Cs_{0.02}FA_{0.98}PbI_3)_{0.97}(MAPbBr_3)_{0.03}$/Spiro-OMeTAD/Au	1.10	25.05	78.2	21.90	150	Non-hydrolytic sol-gel reaction	Perovskite seeding growth	[S9]
FTO/TiO_2/$MAPb_{0.9}Sn_{0.05}Cu_{0.05}I_{2.9}B_{r0.1}$/Spiro-OMeTAD/$MoO_3$/Ag	1.08	23.97	81.0	21.08	70	Chemical bath deposition	Pb-Sn-Cu ternary perovskite	[S10]
ITO/am-TiO_2/brookite TiO_2/$MAPbI_3$/Spiro-OMeTAD/Au	1.16	22.40	83.0	21.60	150	Brookite TiO_2 slurry (purchase)	Brookite TiO_2 as ETL	[S11]
FTO/SnO_2@TiO_2/$(FAPbI_3)_{0.3}(MAPbI_3)_{0.7}$/Spiro-OMeTAD/Au	1.11	24.56	80.5	21.96	140	Chemical bath deposition	SnO_2-modified TiO_2 as ETL	[S12]
ITO/TiO_2-Cl/ $MA_{0.03}FA_{0.97}Pb(I_{0.97}Br_{0.03})_3$/Spiro-OMeTAD/Au	1.12	24.30	81.1	22.10	140	Nonhydrolytic sol-gel reaction	CsCl-enhanced PbI_2 precursor	[S13]
ITO/TiO_2-Cl/$FAPbI_3$-based perovskite/Spiro-OMeTAD/Au	1.15	24.80	78.4	22.30	140	Nonhydrolytic sol-gel reaction	Double-side-passivation by PbI_2	[S14]
ITO/NH_2-TiO_2/$Cs_{0.05}FA_{0.83}MA_{0.12}PbI_{2.55}Br_{0.45}$/Spiro-OMeTAD/Au	1.19	23.40	76.6	21.33	150	Nonhydrolytic sol-gel reaction	Amino functionalization of TiO_2	[S15]
FTO/TiO_2/$Cs_{0.06}FA_{0.82}MA_{0.12}PbI_3$:P(VDF-TrFE)/P(VDF-TrFE)/Spiro-OMeTAD/MoO_3/Ag	1.18	23.76	78.0	21.76	——	Chemical bath deposition	Polarize ferroelectric polymers	[S16]
FTO/Eu-TiO_2/$(Cs_{0.05}FA_{0.80}MA_{0.15})Pb(I_{0.85}Br_{0.15})_3$/Spiro-OMeTAD/Au	1.13	23.85	79.0	21.40	100	Chemical-bath deposition	Europium-doped Titania	[S17]

FTO/TiO_2 colloids/$FAPbI_3$+$MDACl_2$+MACl/Spiro-OMeTAD/Au	1.13	25.23	79.0	22.70	100	Spray pyrolysis method	TiO_2 Colloid-Spray Coating	[S18]
ITO/TiO_2-Cl/$Cs_{0.05}FA_{0.9}MA_{0.05}PbI_{2.85}Br_{0.15}$/Spiro-OMeTAD/Au	1.15	24.3	81.4	22.70	150	Nonhydrolytic sol-gel reaction	Cross-linked grain encapsulation	[S19]
ITO/F-TiO_2 /$K_{0.025}Cs_{0.05}FA_{0.83}MA_{0.12}PbI_{2.55}Br_{0.45}$ /Spiro-OMeTAD/Au	**1.21**	**23.36**	**80.3**	**22.68**	**150**	**Nonhydrolytic sol-gel reaction**	**Fluorine Functionalization of TiO_2**	**This work**

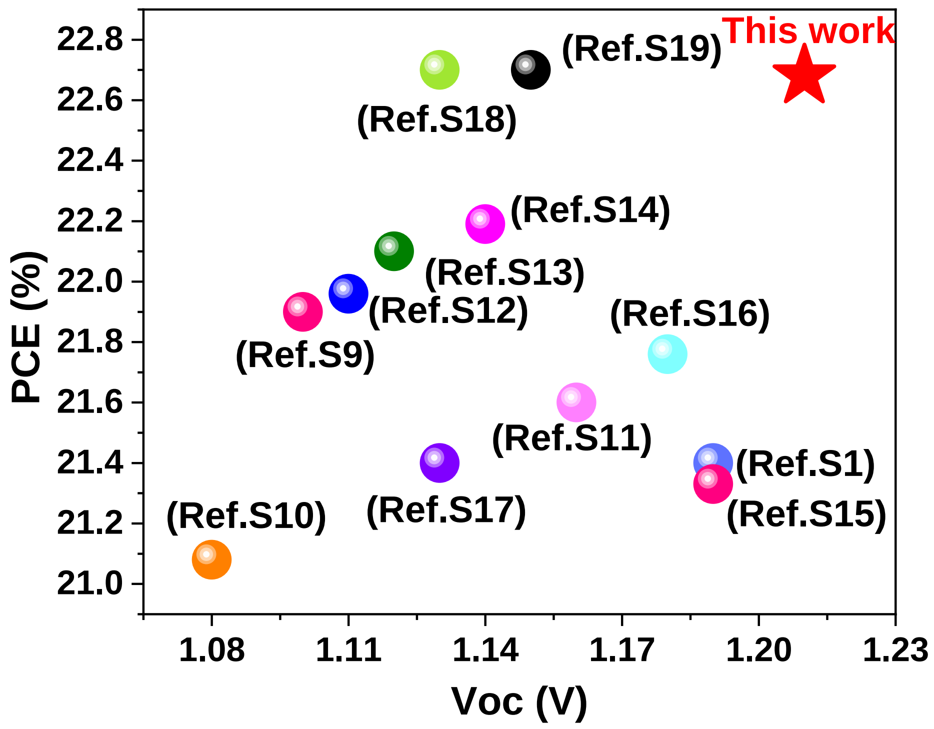

Figure S15. Comparison of the PCE and V_{oc} of devices obtained in this work with those of the PHJ-PSC devices based on low-temperature solution-processed TiO_2 ETLs reported in literatures.

S18. Box charts of the statistical photovoltaic parameters of PSCs based on different TiO_2 ETLs.

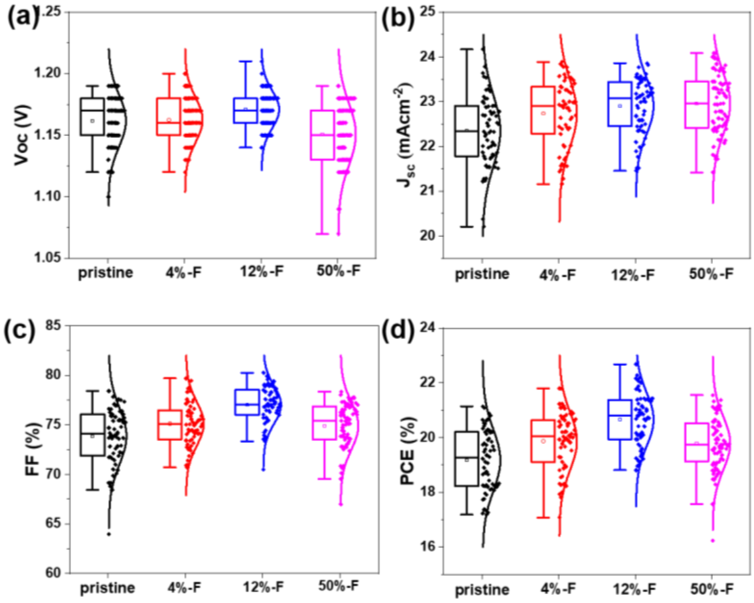

Figure S16. Box charts of the statistical photovoltaic parameters of V_{oc} (a), J_{sc} (b), FF (c) and PCE (d) for over 60 devices based on pristine TiO_2 and F-TiO_2 ETLs.

S19. Photovoltaic parameters of the flexible PHJ-PSC devices based on different TiO_2 ETLs.

Table S8. Photovoltaic parameters of the flexible PHJ-PSC devices based on different TiO_2 ETLs under one sun illumination (AM 1.5G, 100 mA cm^{-2}).

Devices	Molar ratio of F:Ti [a]		V_{oc} (V)	J_{sc} (mA/cm^2)	FF (%)	PCE (%)	R_s [c] (Ω•cm^2)	R_{sh} [c] (Ω•cm^2)
pristine TiO_2	0%	Average [b]	1.13 ±0.02	18.80±1.29	68.09±4.69	14.49±1.87	7.56	2028.74
		Best	1.17	20.19	74.55	17.61	5.84	2456.85
4%-F-TiO_2	4%	Average [b]	1.14 ±0.03	19.10±1.06	69.60±4.75	15.23±1.86	6.87	2173.93
		Best	1.17	19.90	77.94	18.18	4.67	3628.12
12%-F-TiO_2	12%	Average [b]	1.16 ±0.03	19.56±1.19	70.52±3.59	16.01±1.46	6.27	3177.08
		Best	1.19	20.51	75.09	18.26	4.53	8138.83
50%-F-TiO_2	50%	Average [b]	1.12±0.02	19.72±0.98	69.99±4.77	15.54±1.80	6.73	2843.41
		Best	1.13	20.89	77.00	18.21	4.33	3382.56

[a] the molar ratio of F^- relative to Ti^{4+} in the raw solution; [b] Averaged over 20 devices fabricated independently; [c] R_s and R_{sh} are given by the PCE measurement system.

S20. Comparison of PCE and V_{oc} values of the flexible PHJ-PSCs based on LT-TiO_2 ETLs reported in literatures.

Table S9. A list of photovoltaic parameters of the typical flexible PHJ-PSC devices based on LT-TiO_2 ETLs reported in literatures.

Structure	V_{oc} (V)	J_{sc} (mAcm^{-2})	*FF* (%)	PCE (%)	Tempreture (°C)	Method of TiO_2 preparation	Ref.
PET/ITO/TiO_x/$MAPbI_3$:C-PCBOD/Spiro-OMeTAD/MoO_3/Ag	1.01	21.62	76.0	18.10	70	Chemical bath deposition	[S20]
PEN/ITO/TiO_x/NH_4Cl/CsFAMA perovskite/Spiro-OMeTAD/Au	1.12	22.45	70.6	17.69	100	Nonhydrolytic sol-gel reaction	[S21]
PETUG/C_{60}/u-TiO_x/$FA_{0.85}MA_{0.15}PbI_{2.55}Br_{0.45}$/Spiro-OMeTAD/Au	1.04	19.60	72.3	14.73	<70	Chemical bath deposition	[S22]
PET/ITO/e-beam TiO_2/$MAPbI_{3-x}Cl_x$/PTAA/Au	0.91	21.30	69.0	13.50	<77	Electron beam evaporation	[S23]
PET/ITO/TiO_2/$MAPbI_{3-x}Cl_x$/PTAA/Au	1.11	20.77	69.0	15.88	Room temperature	Magnetron sputtering	[S24]
PEN/ITO/UV-Nb-TiO_2/$MAPbI_3$/Spiro-OMeTAD/Au	1.04	20.20	76.0	16.01	<50	Nonhydrolytic sol-gel reaction	[S25]
PEN/ITO/TiO_2-C_{60}/$(FAPbI_3)_x(MAPbCl_3)_{1-x}$/Spiro-OMeTAD/Ag	1.01	23.54	69.0	16.39	120	Hydrolytic sol-gel reaction	[S26]
PEN/ITO/TiO_2/$MAPbI_3$/Spiro-OMeTAD/Ag	1.00	20.04	71.0	14.30	Room temperature	Hydrolytic sol-gel reaction	[S27]
PEN/ITO/c-TiO_2/RIE-mp-TiO_2/$(FAPbI_3)_{0.97}(MAPbBr_3)_{0.03}$/Spiro-OMeTAD/Au	1.07	22.11	73.0	17.29	150	Reactive ion etching (RIE) method	[S28]

PET/ITO/R-Fu/Lt-TiO_2 nanocomposite/$(FAI)_{0.85}$ $(PbI_2)_{0.89}(MABr)_{0.15}(PbBr_2)_{0.15}$ /Spiro-OMeTAD/MoO_3/Ag	1.05	22.93	75.0	18.06	100	Nanoparticle ball milling method	[S29]
PEN/ITO/F-TiO_2/KCsFAMA perovskite/Spiro-OMeTAD /Au	**1.19**	**20.51**	**75.1**	**18.26**	**150**	**Nonhydrolytic sol-gel reaction**	**This work**

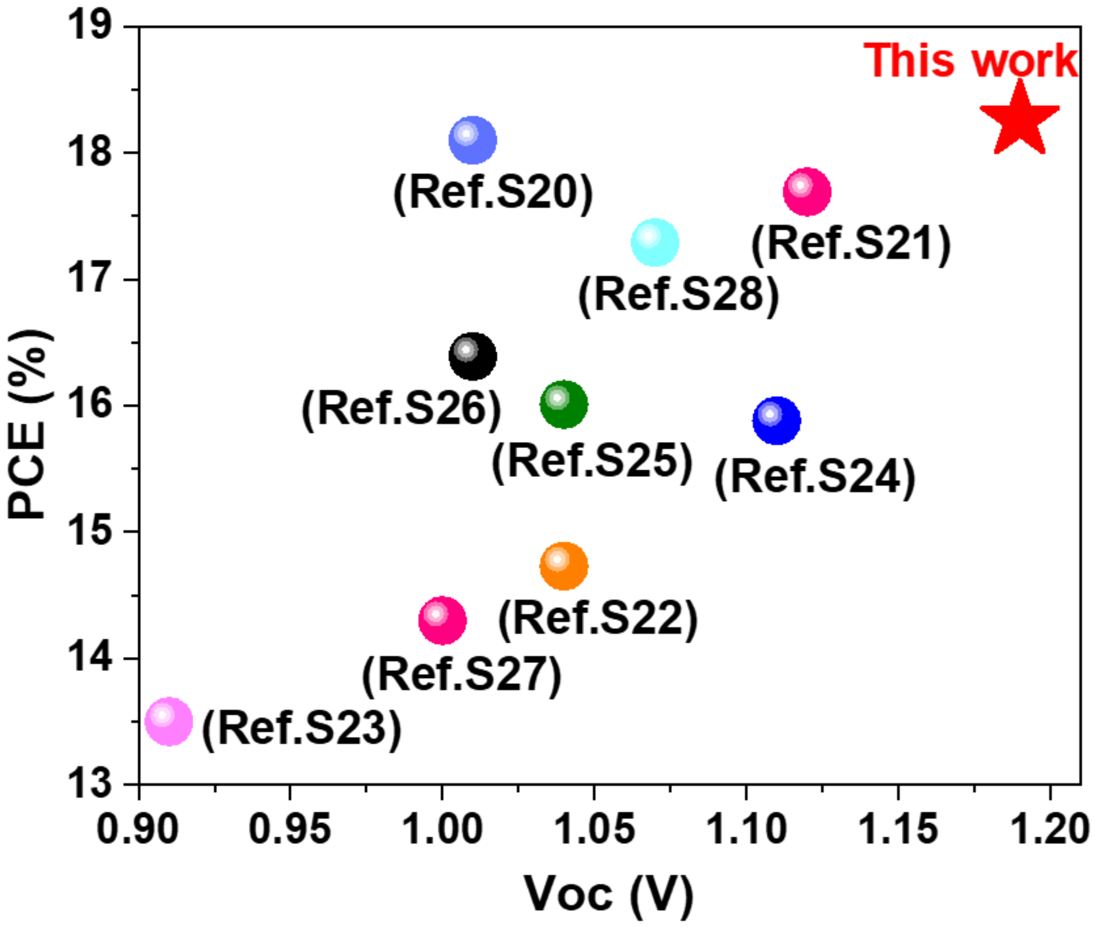

Figure S17. Comparison of the PCE and V_{oc} of devices obtained in this work with the typical flexible perovskite solar cells based on LT-TiO_2 ETLs reported in literatures.

S21. Work functions of different TiO_2 ETLs and CsFAMA perovskite film.

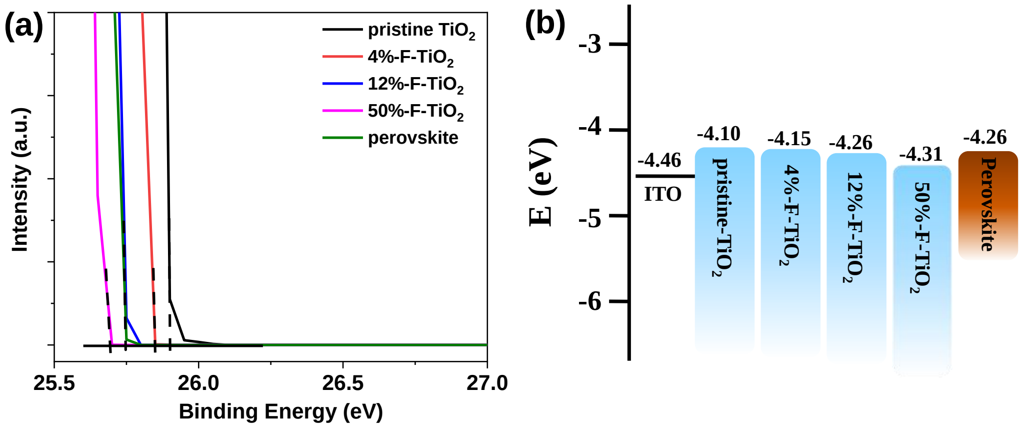

Figure S18. (a) UPS spectra of different TiO_2 and perovskite films. (b) Energy level diagram for the corresponding materials used in our PSC devices measured by UPS. The energy level of ITO was referenced in the literature [S30].

S22. Electron trap-state densities of perovskite films deposited on different TiO_2 ETLs.

The electron trap-state density within the perovskite layer is measured by using space charge limited current (SCLC) method. An electron-only device with a structure of ITO/F-TiO_2/KCsFAMA perovskite/PCBM/Ag was fabricated, and the corresponding I-V curves for the devices based on the 4%-F-TiO_2, 12%-F-TiO_2, 50%-F-TiO_2 and pristine TiO_2 ETLs are presented in Figure S19. An ohmic response is emerged at the low bias. When the voltage increase and exceed the kink-point voltage, the applied voltage at the kink-point voltage is defined as the trap-filled limit voltage (V_{TFL}). The onset voltage V_{TFL} is linearly proportional to the density of trap states n_t according to equation (S1):

$$n_t = \frac{2\varepsilon\varepsilon_0}{eL^2} V_{TFL}$$

(S1)

where e is the elementary charge of the electron (e = 1.6 $\times 10^{-19}$ C), d is the perovskite film thickness, ε is the relative dielectric constant of perovskite ($FAPbI_3$ = 46.9), ε_0 is the vacuum permittivity (ε_0 = 8.854 $\times 10^{-12}$ F/m), and n_t is the trap state density of different treated films. The measured trap-state densitys (n_t) of perovskite films based on different TiO_2 ETLs are listed in Table S10.

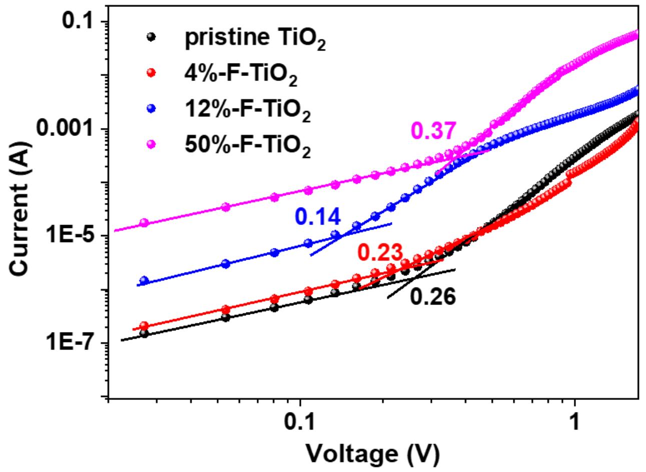

Figure S19. Dark current-voltage responses of the electron-only devices based on different TiO_2 ETLs, from which the trap-filled limit voltage (V_{TFL}) is determined as the kink point.

Table S10. The measured electron trap-state density (n_t) of perovskite films based on different TiO_2 ETLs.

Device	L (nm)	V_{TFL} (V)	n_t (cm^{-3})
pristine-TiO_2	425	0.26	6.67×10^{15}
4%-F-TiO_2	425	0.23	5.90×10^{15}
12%-F-TiO_2	425	0.14	$3.59\times10_{15}$
50%-F-TiO_2	425	0.37	9.49×10^{15}

S23. Deep level defects of devices based on different TiO_2 ETLs estimated by DLTS.

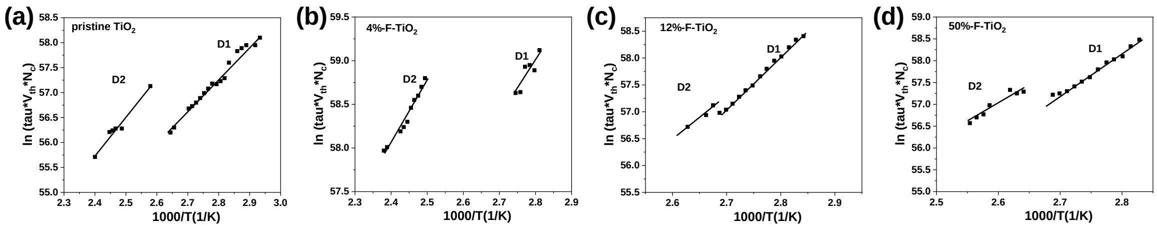

Figure S20. Arrhenius plots of devices based on different TiO_2 ETLs obtained from DLTS spectra. (a) pristine TiO_2, (b) 4%-F-TiO_2, (c) 12%-F-TiO_2 and (d) 50%-F-TiO_2.

Table S11. The defect parameters of devices based on different TiO_2 ETLs.

Defects		E_T [a] (eV)	Sigma[b] (cm^2)	N_T [c] (cm^{-3})	N_A [d] (cm^{-3})
Control	D1	E_V+0.557	9.35×10^{-18}	9.72×10^{14}	1.52×10^{16}
	D2	E_V+0.656	5.29×10^{-17}	5.71×10^{14}	
4%-F-TiO_2	D1	E_V+0.606	8.31×10^{-18}	6.69×10^{14}	1.61×10^{16}
	D2	E_V+0.659	5.91×10^{-18}	4.01×10^{14}	
12%-F-TiO_2	D1	E_V+0.839	4.31×10^{-14}	4.75×10^{14}	1.45×10^{16}
	D2	E_V+0.706	6.33×10^{-15}	2.20×10^{14}	
50%-F-TiO_2	D1	E_V+0.800	1.08×10^{-14}	3.82×10^{14}	1.62×10^{16}
	D2	E_V+0.754	1.32×10^{-15}	6.30×10^{14}	

[a]E_T : the energy level of defect; [b] Capture cross-section : capture cross-sections of hole traps; [c] N_T : trap concentration. [d] N_A: the net carrier concentration in film. $N_T = \frac{2\Delta C_{max}}{C_0} N_A$

DLTS uses the transient capacitance of p-n junction at different temperature as a probe to monitor the changes in charge state of a deep defect center.[S31] Traps in the device are filled by carriers through applying a voltage pulse to the device, which changes the capacitance associated with p-n junction of the device.[S32] The types of traps can be differentiated by the change of capacitance during the discharging process of traps (the hole and electron traps correspond to negative and positive ΔC, respectively in n-type films). The activation energies (E_C-E_T or E_T-E_V) and capture cross-section of traps obtained from Arrhenius plots in Figure S20 are summarized in Table S11.

S24. Atomic structures and projected density of states of the main antisite defects of perovskite before and after F incorporation.

Computational details: To investigate the influence of F anions on the surface of $FAPbI_3$ perovskite, we constructed the slab models of $FAPbI_3$. The cell parameters of optimized bulk $FAPbI_3$ structure are a=b=3.3613Å, c=6.3613Å with a simple cubic phase, similar to the experimental values.[S33] And the perovskite slab consists of 8 $FAPbI_3$ units with (001) exposed face and a vacuum 15Å was used to avoid the interactions between periodically repeated slabs. It is worth to mention that the bottom two layers atoms (one layer is the I-FA, another layer is Pb-I) were fixed while the rest of atoms were relaxed during all calculations. The geometry optimization of slab was performed using an 2x2x1 Monkhorst-Pack k-point mesh, while the electronic structure calculations were carried out with a denser 4x4x1mesh.[S34] We chose the PbI_2-terminated surface for our study instead of the FAI-terminated surface, because of the former has lower energy whether in the perovskite surface or the $FAPbI_3$@TiO_2 interface. Four surface antisite defect structures were considered: lead antisite (Pb_I, corresponding to I site substituted by Pb), iodine antisite (I_{Pb}, corresponding to Pb site substituted by I), fluorine replacing iodine (F_I) and fluorine replacing lead (F_{Pb}).The defect formation energies were obtained as:[S35]

$$\mathbf{E}_{\boldsymbol{f}} = \mathbf{E}_{\boldsymbol{defect}} - \mathbf{E}_{\boldsymbol{perfect}} + \sum_{\boldsymbol{i}} \boldsymbol{n_i E_i}$$

Where $\mathbf{E}_{\boldsymbol{defec}}$ and $\mathbf{E}_{\boldsymbol{perfect}}$ is the total energy of the surface with an without a defect respectively, $\mathbf{n}_i$ is the number of atom removed ($\boldsymbol{i}$>0) from or added ($\boldsymbol{i}$<0) to the surface and $\mathbf{E}_{\boldsymbol{i}}$ is the DFT-calculated energy of the element.

DFT calculations were performed to examine the influence of F anions on the surface of $FAPbI_3$ perovskite. Because the band edges of perovskite were reported to be composed of Pb and I orbitals[S36], we just taken the Pb and I involving defects into consideration, including vacancy defects and I-Pb antisite defects. Regarding to

the vacancy defects, Li and his coworkers[S37] have confirmed that the fluoride ions are very effective in passivating both the organic cation and halide anion vacancies by forming strong hydrogen bonds with organic cations (FA) and strong ionic bonds with lead in the perovskite films**.** Therefore, we just focused on the impact of the fluoride ions to the antisite defects of perovskite surface. As shown in Figure S21(c,d) and (e,f), the Pb-I antisite defects can easily result in formation of strong covalent bonds and lead to localized traps state within the band gap which will serve as the charge recombination centers. when incorporated into the TiO_2/perovskite interface, fluoride ions can easily replace the iodine ions to form strong ionic bonds with lead on the perovskite surface owing to the lowest defect formation energy (Table S12), which is consistent with the previous research [S37].More interesting, there is no obvious localized traps state in the bandgap of perovskite according to the analysis of the electronic structure (Figure S21g, h). Although the structure of lead ions replaced by fluoride ions (F_{Pb}) leads local traps state (Figure S21i, j) within the band gap, F_{Pb} defect is more difficult to form than I_{Pb} defect with the higher formation energy (3.022eV for F_{Pb}, 2.869eVv for I_{Pb}) (Table S12), suggesting that the introducing of fluorine can effectively suppress the Pb-I antisite defect. In addition, F_{Pb} defect energy levels tend to be in the middle of the perovskite band gap compared to the I_{Pb} defect energy levels (Figure S21), suggesting F_{Pb} defect has the higher activation energies than I_{Pb} defect, which causes the D_1 defect to move gradually to the higher temperature region.

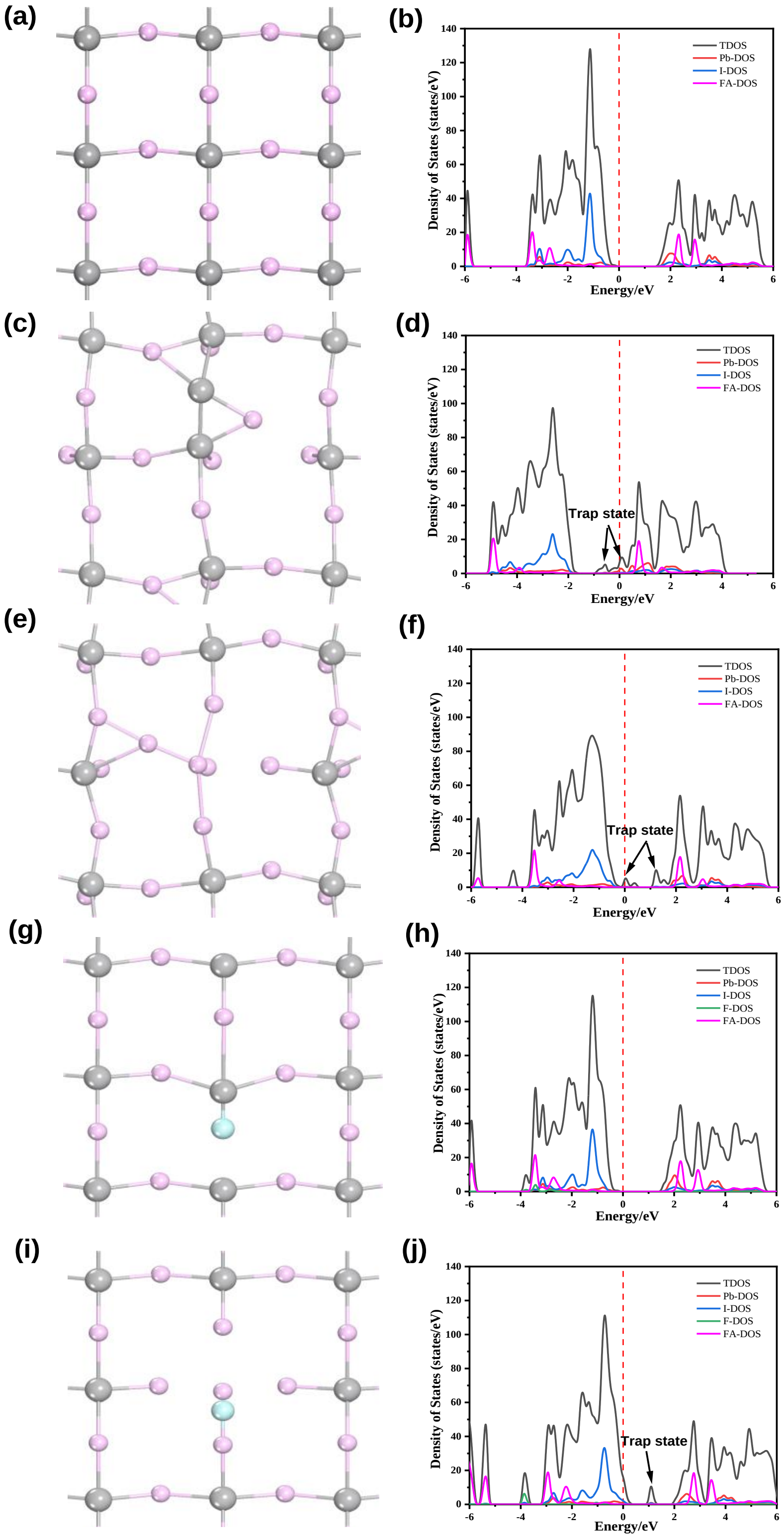

Figure S21. Atomic structure and project density of states of the perfect $FAPbI_3$ (a,b) and four main defects located on the surface of $FAPbI_3$: (c,d) Pb_I, (e,f) I_{Pb}, (g,h) F_I, (i,j) F_{Pb}. The gray, pink and cyan spheres denote the Pb, I, F ions respectively, and organic FA ions are hidden away for

easier observation.

Table S12. The calculated defect formation energies ($E_{form.}$) on the surface of $FAPbI_3$ perovskite.

Defect type	$E_{form.}$ (eV)
Pb_I	0.414
I_{Pb}	2.869
F_I	-2.085
F_{Pb}	3.022

S25. Analysis of time-resolved photoluminescence (TRPL) spectra of the perovskite films deposited on different TiO_2 ETLs.

To gain a further understanding of the steady-state PL results, we measured the time-resolved photoluminescence (TRPL) spectra of the KCsFAMA perovskite film based on different TiO_2 (Figure 4c). The excitation source was a 543 nm picosecond laser pulse which was filtered from a supercontinuum generation. The TRPL spectrum can be fitted by a bi-exponential decay function as shown in equation (S2):[S38]

$$f(t) = A_1 \cdot \exp\left(\frac{-t}{\tau_1}\right) + A_2 \cdot \exp\left(\frac{-t}{\tau_2}\right) + B \qquad \text{(S2)}$$

Where A_1, A_2, τ_1, τ_2 and B are the decay amplitude, the decay lifetime, and a constant for the baseline offset, respectively. The fast decay lifetime, τ_1, indicates that the PL quenching originated from the TiO_2/perovskite interfacial charge transfer; while the slow decay lifetime, τ_2, represented the nonradiative recombination of trapped charges within perovskite layers. The average decay time (τ_{ave}) of the perovskite/TiO_2 films were calculated according to the formula $\tau_{ave} = (A_1\tau_1^2 + A_2\tau_2^2)/(A_1\tau_1 + A_2\tau_2)$ [S39]. The average decay time (τ_{ave}) of the perovskite/TiO_2 films are listed in Table S13.

Table S13. Time constants in TRPL determined by bi-exponential fittings measured on perovskite films based on different TiO_2 films.

Sample	A1	τ1 (ns)	A2	τ2 (ns)	t (ns)
pristine TiO_2	0.09	8.29	0.67	253.99	252.92
4%-F-TiO_2	0.06	7.83	0.67	268.65	267.97
12%-F-TiO_2	0.11	7.69	0.66	248.11	246.87
50%-F-TiO_2	0.20	7.44	0.58	221.96	219.51

S26. Fitting parameters for EIS data.

The influence of F-TiO_2 on the interfacial charge transport behavior of the devices was investigated by using the electrochemical impedance spectroscopy (EIS).[S40] The Nyquist plots of devices based on different TiO_2 ETLs measured in the dark under a reverse potential of 1.0 V (near the open circuit potential) are compared in Figure 4f. Based on the equivalent circuit model, the data in the Nyquist plot is separated into two R-CPE arcs, consisting of a resistor R_s (series resistance, which is defined as the starting point at the real part of the Nyquist plot) connected with two parallel R-CPE elements. The R_{co} (contact resistance) is characterized by the high-frequency feature, which is associated with the ETL/perovskite interface. The recombination resistance (R_{rec}, which is sensitively dependent on the recombination properties) and the non-ideal chemical capacitances (*CPE1*, *CPE2*) of the system are determined by the lower frequency element.[S15] The parameters used for fitting are summarized in Table S14.

Table S14. Parameters employed for the fitting of the impedance spectra of devices based on different TiO_2 ETLs.

Device	R_s (Ω)	R_{co} (Ω)	CPE1-T (F)	CPE1-P (F)	R_{rec} (Ω)	CPE2-T (F)	CPE2-P (F)
pristine-TiO_2	25.46	168	5.30E-07	1.20	5690	1.39E-07	0.96
4%-F-TiO_2	8.2	48	9.99E-07	1.00	7900	9.69E-9	0.96

12%-F-TiO_2	5.56	25	7.04E-08	0.95	8420	2.79E-08	0.95
50%-F-TiO_2	6.28	40	1.14E-07	1.20	7790	1.87E-08	0.94

S27. Ambient stabilities of the devices based on different TiO_2 ETLs.

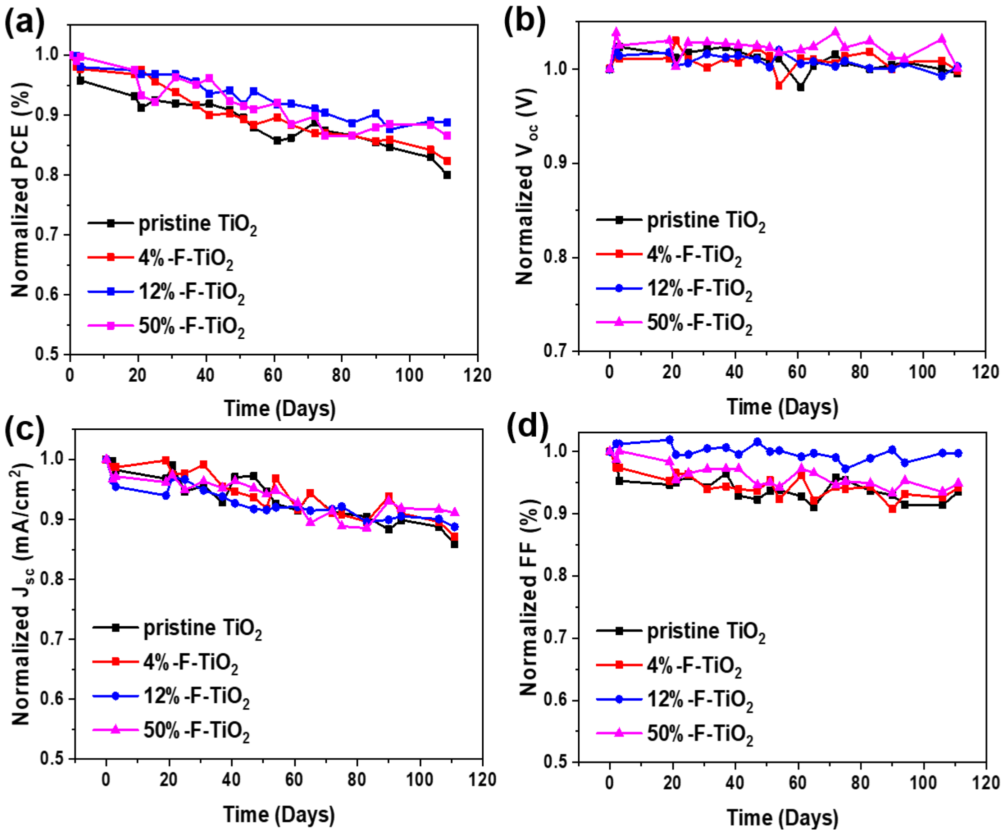

Figure S22. Ambient stabilities of the un-encapsulated devices based on different TiO_2 ETLs measured in ambient condition (temperature: 25 °C, relative humidity: 35%) for 110 d: (a) PCE, (b) V_{oc}, (c) J_{sc} and (d) FF.

Reference

[S1] a) G. Kresse, J. Furthmuller, *Phys. Rev. B* **1996**, *54*, 11169; b) G. Kresse, J. Furthmuller, *Comput. Mater. Sci.* **1996**, *6*, 15.

[S2] a) P. E. Blöchl, *Phys. Rev. B* **1994**, *50*, 17953; b) G. Kresse, D. Joubert, *Phys. Rev. B* **1999**, *59*, 1758.

[S3] J. P. Perdew, K. Burke, M. Ernzerhof, *Phys. Rev. Lett.* **1996**, *77*, 3865.

[S4] S. Grimme, S. Ehrlich, L. Goerigk, *J. Comput. Chem.* **2011**, *32*, 1456.

[S5] P. A. Giuseppe Mattioli, Francesco Filippone, Ruggero Caminiti, Aldo Amore Bonapasta, *J. Phys. Chem. C* **2010**, *114*, 21694.
[S6] Dudarev, *Phys. Rev. B* **1998**, *57*, 1505
[S7] K. Maeda, K. Domen, *J. Phys. Chem. C* **2007**, *111*, 7851 .
[S8] H. Tan, A. Jain, O. Voznyy, X. Lan, F. P. Garcia de Arquer, J. Z. Fan, R. Quintero-Bermudez, M. Yuan, B. Zhang, Y. Zhao, *Science* **2017**, *355*, 722.
[S9] Y. Zhao, H. Tan, H. Yuan, Z. Yang, J. Z. Fan, J. Kim, O. Voznyy, X. Gong, L. N. Quan, C. S. Tan, *Nat. Commun.* **2018**, *9*, 1607.
[S10] M. Li, Z. K. Wang, M. P. Zhuo, Y. Hu, K. H. Hu, Q. Q. Ye, S. M. Jain, Y. G. Yang, X. Y. Gao, L. S. Liao, *Adv. Mater.* **2018**, *30*, 1800258.
[S11] A. Kogo, Y. Sanehira, Y. Numata, M. Ikegami, T. Miyasaka, *ACS Appl. Mater. Interfaces* **2018**, *10*, 2224.
[S12] B. Ding, S.Y. Huang, Q.Q. Chu, Y. Li, C.X. Li, C.J. Li, G.J. Yang, *J. Mater. Chem. A* **2018**, *6*, 10233.
[S13] Q. Li, Y. Zhao, R. Fu, W. Zhou, Y. Zhao, X. Liu, D. Yu, Q. Zhao, *Adv. Mater.* **2018**, *30*, 1803095.
[S14] Y. Zhao, Q. Li, W. Zhou, Y. Hou, Y. Zhao, R. Fu, D. Yu, X. Liu, Q. Zhao, *Solar RRL* **2019**, *3*, 1800296.
[S15] W. Hu, W. Zhou, X. Lei, P. Zhou, M. Zhang, T. Chen, H. Zeng, J. Zhu, S. Dai, S. Yang, *Adv. Mater.* **2019**, *31*, 1806095.
[S16] C. C. Zhang, Z. K. Wang, S. Yuan, R. Wang, M. Li, M. F. Jimoh, L. S. Liao, Y. Yang, *Adv. Mater.* **2019**, *31*, 1902222.
[S17] P. Chen, Z. Wang, S. Wang, M. Lyu, M. Hao, M. Ghasemi, M. Xiao, J.H. Yun, Y. Bai, L. Wang, *Nano Energy* **2020**, *69*, 104392.
[S18] M. J. Paik, Y. Lee, H.-S. Yun, S.-U. Lee, S.-T. Hong, S. I. Seok , *Adv. Energy Mater.* **2020**, 2001799.
[S19] K. Xiao, Q. Han, Y. Gao, S. Gu, X. Luo, R. Lin, J. Zhu, J. Xu, Hairen Tan. *J. Energy Chem.* **2021**, *56*, 455.)
[S20] M. Li, Y. G. Yang, Z. K. Wang, T. Kang, Q. Wang, S. H. Turren- Cruz, X. Y. Gao, C. S. Hsu, L. S. Liao, A. Abate, *Adv. Mater.* **2019**, *31*, 1901519.
[S21] J. Jiang, X. Jia, S. Wang, Y. Chen, W. Liu, J. Ding, N. Yuan, *ChemSusChem* **2018**, *11*, 4131.
[S22] C. Liu, M. Cai, Y. Yang, Z. Arain, Y. Ding, X. Shi, P. Shi, S. Ma, T. Hayat, A. Alsaedi, *J. Mater. Chem. A* **2019**, *7*, 11086.
[S23] W. Qiu, U. W. Paetzold, R. Gehlhaar, V. Smirnov, H. G. Boyen, J. G. Tait, B. Conings, W. Zhang, C. B. Nielsen, I. McCulloch, *J. Mater. Chem. A* **2015**, *3*, 22824.
[S24] S. S. Mali, C. K. Hong, A. I. Inamdar, H. Im, S. E. Shim, *Nanoscale* **2017**, *9*, 3095.
[S25] I. Jeong, H. Jung, M. Park, J. S. Park, H. J. Son, J. Joo, J. Lee, M. J. Ko, *Nano Energy* **2016**, *28*, 380.
[S26] Y. Q. Zhou, B. S. Wu, G. H. Lin, Z. Xing, S. H. Li, L. L. Deng, D. C. Chen, D. Q. Yun, S.Y. Xie, *Adv. Energy Mater.* **2018**, *8*, 1800399.
[S27] X. Deng, G. C. Wilkes, A. Z. Chen, N. S. Prasad, M. C. Gupta, J. J. Choi, *J. Phys. Chem. Lett.* **2017**, *8*, 3206.
[S28] B. J. Kim, S. L. Kwon, M. C. Kim, Y. U. Jin, D. G. Lee, J. B. Jeon, Y. Yun, M. Choi, G.

Boschloo, S. Lee, *ACS Appl. Mater. Interfaces* **2020**, *12*, 7125.

[S29] P. C. Wang, V. Govindan, C. H. Chiang, C. G. Wu, *Solar RRL* **2020**, 2000247.

[S30] W. Zhou, P. Zhou, X. Lei, Z. Fang, M. Zhang, Q. Liu, T. Chen, H. Zeng, L. Ding, J. Zhu, S. Dai, S. Yang, *ACS Appl. Mater. Interfaces* **2018**, *10*, 1897.

[S31] X. Wen, C. Chen, S. Lu, K. Li, R. Kondrotas, Y. Zhao, W. Chen, L. Gao, C. Wang, J. Zhang, *Nat. Commun.* **2018**, *9*, 1.

[S32] T. P. Nguyen, *Phys. Status Solidi (a)* **2008**, *205*, 162.

[S33] N. Liu, C. Yam, *Phys. Chem. Chem. Phys.* **2018**, *20*, 6800.

[S34] H. J. Monkhorst, J. D. Pack, *Phys. Rev. B* **1976**, *13*, 5188.

[S35] H. Chen, Q. Wei, M. I. Saidaminov, F. Wang, A. Johnston, Y. Hou, Z. Peng, K. Xu, W. Zhou, Z. Liu, *Adv. Mater.* **2019**, *31*, 1903559.

[S36] Z. Xiao, Z. Song, Y. Yan, *Adv. Mater.* **2019**, *31*, 1803792.

[S37] N. Li, S. Tao, Y. Chen, X. Niu, C. K. Onwudinanti, C. Hu, Z. Qiu, Z. Xu, G. Zheng, L. Wang, *Nat. Energy* **2019**, *4*, 408.

[S38] B. Li, J. Zhen, Y. Wan, X. Lei, L. Jia, X. Wu, H. Zeng, M. Chen, G.W. Wang, S. Yang,*J. Mater. Chem. A* **2020**, *8*, 3872.

[S39] L. Jia, B. Li, Y. Shang, M. Chen, G.-W. Wang, S. Yang, *Org. Electron.* **2020**, *82*.105726

[S40] W. Zhou, D. Li, Z. Xiao, Z. Wen, M. Zhang, W. Hu, X. Wu, M. Wang, W. H. Zhang, Y. Lu, *Adv. Funct. Mater.* **2019**, *29*, 1901026.
